# Post-COVID-19-associated morbidity in children, adolescents, and adults: A matched cohort study including more than 157,000 individuals with COVID-19 in Germany

**DOI:** 10.1371/journal.pmed.1004122

**Published:** 2022-11-10

**Authors:** Martin Roessler, Falko Tesch, Manuel Batram, Josephine Jacob, Friedrich Loser, Oliver Weidinger, Danny Wende, Annika Vivirito, Nicole Toepfner, Franz Ehm, Martin Seifert, Oliver Nagel, Christina König, Roland Jucknewitz, Jakob Peter Armann, Reinhard Berner, Marina Treskova-Schwarzbach, Dagmar Hertle, Stefan Scholz, Stefan Stern, Pedro Ballesteros, Stefan Baßler, Barbara Bertele, Uwe Repschläger, Nico Richter, Cordula Riederer, Franziska Sobik, Anja Schramm, Claudia Schulte, Lothar Wieler, Jochen Walker, Christa Scheidt-Nave, Jochen Schmitt

**Affiliations:** 1 Center for Evidence-Based Healthcare (ZEGV), University Hospital Carl Gustav Carus and Carl Gustav Carus Faculty of Medicine, TU Dresden, Dresden, Germany; 2 Vandage GmbH, Bielefeld, Germany and Faculty for Business Administration and Economics, Bielefeld University, Bielefeld, Germany; 3 InGef—Institute for Applied Health Research Berlin, Berlin, Germany; 4 Techniker Krankenkasse, Hamburg, Germany; 5 AOK Bayern—Die Gesundheitskasse, Regensburg, Germany; 6 BARMER Institut für Gesundheitssystemforschung (bifg), Berlin, Germany; 7 Department of Pediatrics, University Hospital Carl Gustav Carus and Carl Gustav Carus Faculty of Medicine, TU Dresden, Dresden, Germany; 8 Robert Koch-Institute, Berlin, Germany; 9 AOK PLUS, Dresden, Germany; 10 DAK-Gesundheit, Hamburg, Germany; London School of Hygiene and Tropical Medicine, UNITED KINGDOM

## Abstract

**Background:**

Long-term health sequelae of the Coronavirus Disease 2019 (COVID-19) are a major public health concern. However, evidence on post-acute COVID-19 syndrome (post-COVID-19) is still limited, particularly for children and adolescents. Utilizing comprehensive healthcare data on approximately 46% of the German population, we investigated post-COVID-19-associated morbidity in children/adolescents and adults.

**Methods and findings:**

We used routine data from German statutory health insurance organizations covering the period between January 1, 2019 and December 31, 2020. The base population included all individuals insured for at least 1 day in 2020. Based on documented diagnoses, we identified individuals with polymerase chain reaction (PCR)-confirmed COVID-19 through June 30, 2020. A control cohort was assigned using 1:5 exact matching on age and sex, and propensity score matching on preexisting medical conditions. The date of COVID-19 diagnosis was used as index date for both cohorts, which were followed for incident morbidity outcomes documented in the second quarter after index date or later.Overall, 96 prespecified outcomes were aggregated into 13 diagnosis/symptom complexes and 3 domains (physical health, mental health, and physical/mental overlap domain). We used Poisson regression to estimate incidence rate ratios (IRRs) with 95% confidence intervals (95% CIs). The study population included 11,950 children/adolescents (48.1% female, 67.2% aged between 0 and 11 years) and 145,184 adults (60.2% female, 51.1% aged between 18 and 49 years). The mean follow-up time was 236 days (standard deviation (SD) = 44 days, range = 121 to 339 days) in children/adolescents and 254 days (SD = 36 days, range = 93 to 340 days) in adults. COVID-19 and control cohort were well balanced regarding covariates. The specific outcomes with the highest IRR and an incidence rate (IR) of at least 1/100 person-years in the COVID-19 cohort in children and adolescents were malaise/fatigue/exhaustion (IRR: 2.28, 95% CI: 1.71 to 3.06, *p* < 0.01, IR COVID-19: 12.58, IR Control: 5.51), cough (IRR: 1.74, 95% CI: 1.48 to 2.04, *p* < 0.01, IR COVID-19: 36.56, IR Control: 21.06), and throat/chest pain (IRR: 1.72, 95% CI: 1.39 to 2.12, *p* < 0.01, IR COVID-19: 20.01, IR Control: 11.66). In adults, these included disturbances of smell and taste (IRR: 6.69, 95% CI: 5.88 to 7.60, *p* < 0.01, IR COVID-19: 12.42, IR Control: 1.86), fever (IRR: 3.33, 95% CI: 3.01 to 3.68, *p* < 0.01, IR COVID-19: 11.53, IR Control: 3.46), and dyspnea (IRR: 2.88, 95% CI: 2.74 to 3.02, *p* < 0.01, IR COVID-19: 43.91, IR Control: 15.27). For all health outcomes combined, IRs per 1,000 person-years in the COVID-19 cohort were significantly higher than those in the control cohort in both children/adolescents (IRR: 1.30, 95% CI: 1.25 to 1.35, *p* < 0.01, IR COVID-19: 436.91, IR Control: 335.98) and adults (IRR: 1.33, 95% CI: 1.31 to 1.34, *p* < 0.01, IR COVID-19: 615.82, IR Control: 464.15). The relative magnitude of increased documented morbidity was similar for the physical, mental, and physical/mental overlap domain. In the COVID-19 cohort, IRs were significantly higher in all 13 diagnosis/symptom complexes in adults and in 10 diagnosis/symptom complexes in children/adolescents. IRR estimates were similar for age groups 0 to 11 and 12 to 17. IRs in children/adolescents were consistently lower than those in adults. Limitations of our study include potentially unmeasured confounding and detection bias.

**Conclusions:**

In this retrospective matched cohort study, we observed significant new onset morbidity in children, adolescents, and adults across 13 prespecified diagnosis/symptom complexes, following COVID-19 infection. These findings expand the existing available evidence on post-COVID-19 conditions in younger age groups and confirm previous findings in adults.

**Trial registration:**

ClinicalTrials.gov https://clinicaltrials.gov/ct2/show/NCT05074953.

## Abstract 2

In a matched cohort study from Germany, including more than 157,000 individuals with COVID-19, Dr. Martin Roessler and colleagues investigate post COVID-19 associated morbidity in children, adolescents, and adults.

## Author summary

**Why was this study done?:**

- Some patients with Coronavirus Disease 2019 (COVID-19) suffer from long-term health problems following the acute phase of COVID-19.
- Evidence on post-acute COVID-19 (post-COVID-19) syndrome is still limited, especially for children and adolescents.

**What did the researchers do and find?:**

- We used comprehensive healthcare data from a sample of almost half of the German population to investigate the risk of post-COVID-19 disease patterns in children, adolescents, and adults.
- We identified all patients with polymerase chain reaction (PCR)-confirmed diagnosis of COVID-19 (157,134 individuals in total, 11,950 children/adolescents and 145,184 adults) and matched them to a control cohort of individuals with identical age and sex, and similar preexisting medical conditions without COVID-19.
- We recorded medical conditions documented by a physician at least 3 months after the date of COVID-19 diagnosis and compared them to the matched controls without COVID-19.
- We observed increased rates of newly diagnosed physical and mental health problems in the COVID-19 group, compared to the control group, which differed according to age.

**What do these findings mean?:**

- Although healthcare utilization may differ between those who have suffered COVID-19 and those who have not, the results of our study indicate that people of all age groups (children, adolescents, and adults) are at risk of post-COVID-19 syndrome and that the spectrum of health problems differs between age groups.

## Introduction

There is mounting evidence that a yet unknown proportion of persons suffers from long-term complications after Severe Acute Respiratory Syndrome Coronavirus 2 (SARS-CoV-2) infection. Early in the pandemic, patients started to share their experiences in the social media on what they referred to as “Long-haul COVID” or “Long COVID.” They reported a wide variety of somatic and mental health issues that were either persisting, recurring, or newly occurring beyond the 4-week phase of acute Coronavirus Disease 2019 (COVID-19) and even mild or asymptomatic SARS-CoV-2 infection [1]. Health organizations and medical societies at the national and international level have taken efforts to systemize observations from an increasing body of observational research studies, in order to provide a pragmatic case definition for this new health phenomenon. A World Health Organization (WHO) working group including patients and a multidisciplinary team of medical experts proposed a preliminary clinical case definition using the term “post-COVID-19 condition.” Based on a structured consensus process, this definition refers to a broad spectrum of otherwise unexplained health conditions that are present 3 months after the onset of symptoms or date of SARS-CoV-2 infection and last for at least 2 months [2]. Notably, it is still uncertain whether the definition applies to adults as well as children and adolescents due to the paucity of available data among younger age groups [2].

An increasing number of studies have examined health sequalae at least 3 months after SARS-CoV-2 infection. The majority of previous studies focused on adults, and less than half of these studies included a control group of persons without documented or clinically suspected SARS-CoV2-infection [3,4]. Symptoms frequently observed to be associated with a history of SARS-CoV-2 infection among adults include, but are not limited to, fatigue, lack of memory and concentration, and respiratory symptoms [5–8]. Children and adolescents seem to be less frequently affected by post-COVID-19 condition, but the paucity of data precludes to draw final conclusions. In addition, long-term sequelae of SARS-Cov-2-infection among children and adolescents may well differ from those among adults [9–13].

The need for considering symptoms as well as medical conditions in multiple organ systems is reflected by the WHO working group definition of post-COVID-19 condition among adults [2] and ongoing work to develop standardized core outcome sets for future research on post-COVID-19 condition among children and adults [14–16]. As symptoms associated with post-COVID-19 condition appear to decline over time, controlled studies based on routine medical data may help to identify long-term health conditions following SARS-CoV-2 infection, provided access to healthcare is equally available to individuals with and without SARS-CoV-2 infection. Few such studies have been conducted to this point and almost none of these studies has included both adults and children [5,6,17–36].

Against that background, we investigated documented long-term morbidity associated with COVID-19 based on routine data from 6 German statutory health insurance organizations covering nearly half of the population in Germany. We hypothesized that SARS-CoV-2 infections induce higher morbidity 3 months after first diagnosis of COVID-19 or later in both children/adolescents and adults compared with controls without previous COVID-19. We expected this higher morbidity to be reflected in more intensive utilization of healthcare services and, hence, medical diagnoses documented by physicians.

## Methods

### Study design

As part of the POINTED program (see Section A in S1 Appendix), we designed a retrospective matched cohort study based on routine statutory health insurance data. The study was based on comprehensive healthcare data covering the period between January 1, 2019 and December 31, 2020. The POINTED consortium started to work on the design of this study in May 2021. All methodological details, including cohort definitions, inclusion and exclusion criteria, selection and operationalizations of outcomes and covariates, and statistical methodology, were discussed and documented on an online platform (Confluence) prior to data analysis. There were no data-driven analyses. This study is reported as per the Reporting of studies Conducted using Observational Routinely-collected Data (RECORD) guideline [37] (S1 RECORD Checklist).

### Ethics

The competent authority of the Federal State of Saxony, Germany approved the study protocol and declared waiver of informed consent (reference number: 31–5221.40-15/68). The study was approved by the ethics committee of the TU Dresden (approval number: BO-EK (COVID)-482102021) and adheres to all relevant administrative and legal regulations.

### Data

We used routine data from 6 German statutory health insurance organizations: AOK Bayern—Die Gesundheitskasse, AOK PLUS (analyzed by ZEGV), BARMER, BKKen (analyzed by InGef), DAK Gesundheit (analyzed by Vandage GmbH), and Techniker Krankenkasse. In total, these data cover approximately 38 million persons, which corresponds to 52% of all persons in the German statutory healthcare insurance system (Gesetzliche Krankenversicherung, GKV) and 46% of the total German population. The German GKV provides equal access to healthcare for all insured individuals. This includes free choice of physicians and access to specialist outpatient and inpatient healthcare.

In addition to sociodemographic characteristics (age and sex) and vital status (via the date of death), the data used in our analysis include comprehensive information on healthcare utilization in ambulatory (primary as well as specialist) as well as outpatient and inpatient hospital care. The data include diagnoses (according to the International Statistical Classification of Diseases and Related Health Problems—German Modification, ICD-10-GM) by physicians and psychotherapists, inpatient procedures (according to the “Operationen- und Prozedurenschluessel,” OPS; German modification of the International Classification of Procedures in Medicine, ICPM), outpatient medical procedures (according to “Einheitlicher Bewertungsmassstab,” EBM), and prescribed medications (according to the German Anatomical Therapeutic Chemical (ATC) Classification).

### Post-COVID-19 follow-up

We followed the NICE guideline on long COVID [38] and the clinical case definition of post-COVID-19 condition proposed by WHO [2] and considered an individual to enter the post-COVID-19 phase 3 months after diagnosis of COVID-19. Due to the characteristics of the German healthcare billing system, outpatient diagnoses in our data can be assigned reliably to a specific quarter of the year. Accordingly, we considered a diagnosis to have been made in the post-COVID-19 phase if it was documented in the second quarter after the index date (date of COVID-19 diagnosis) or later (see Section B in S1 Appendix for a graphical illustration). This operationalization ensures a time distance of at least 3 months between date of COVID-19 diagnosis and post-COVID-19 outcome incidence.

### COVID-19 and control cohorts

**Fig 1:**
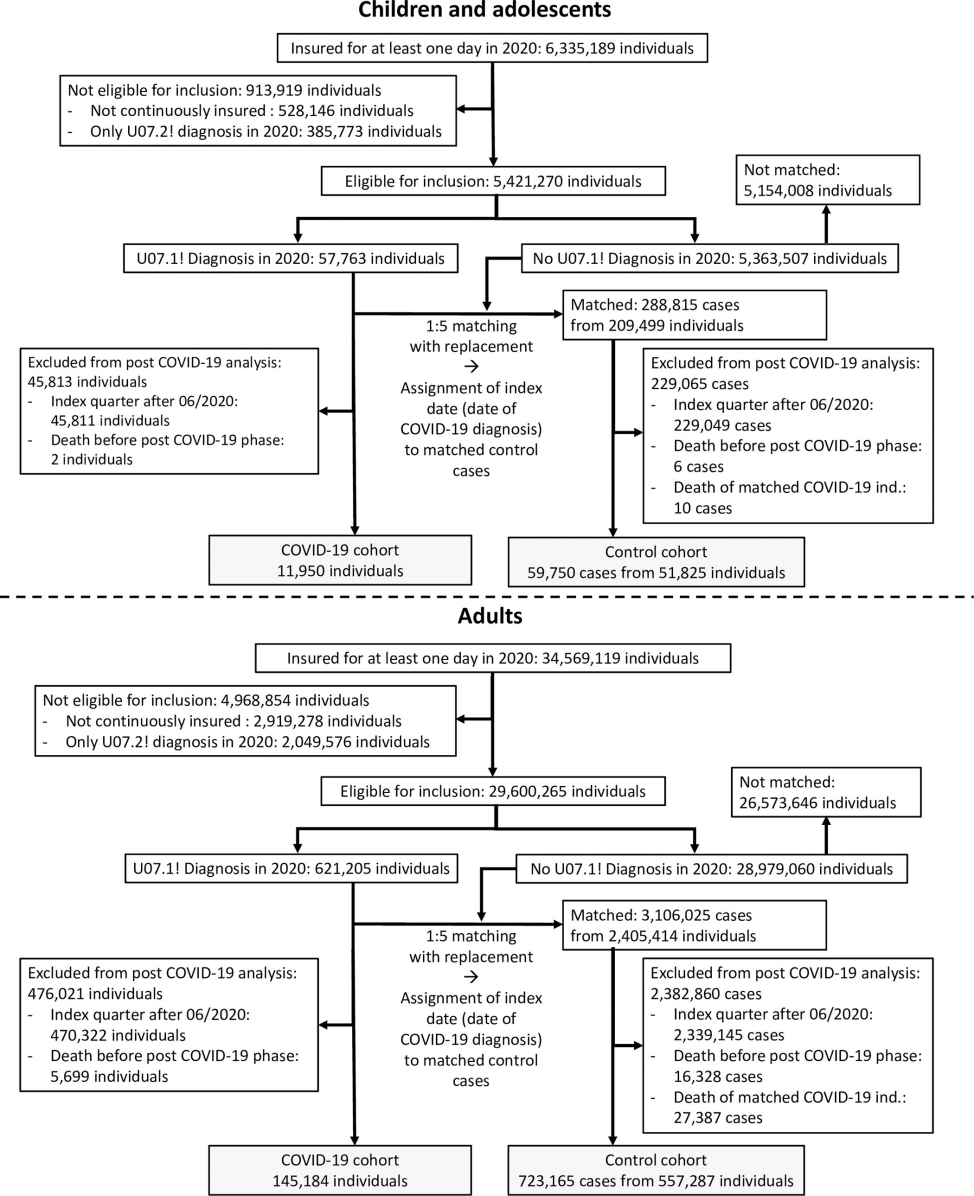
Flow charts for inclusion, exclusion, and matching of children/adolescents and adults with and without COVID-19. The number of individuals insured for at least 1 day in 2020 includes individuals that changed their health insurance organization in 2020. Accordingly, some individuals were represented in multiple datasets of different health insurance organizations. These individuals were excluded because of discontinuous insurance in the first exclusion step and, thus, were not included in our analysis. Exclusion criteria were applied stepwise in the order shown in the flow charts. COVID-19, Coronavirus Disease 2019.

The source population of our study consisted of all individuals insured with one of the 6 considered health insurance organizations for at least 1 day in 2020 (a flow chart depicting inclusion and exclusion criteria is provided in the Results section; see Fig 1). The COVID-19 cohort included individuals with documented COVID-19 diagnosis with polymerase chain reaction (PCR)-based laboratory virus detection (ICD-10-GM: U07.1!) until June 30, 2020. COVID-19 diagnoses could be documented by physicians in both outpatient and inpatient settings. The control cohort included individuals without COVID-19 diagnosis in 2020. To be included in the control cohort, an individual had to be insured with one of the health insurance organizations providing data for our analysis. However, to avoid bias due to selection of controls with increased healthcare needs, individuals were not required to have utilized healthcare services during the observation period to be included in the control cohort. We generally excluded individuals with COVID-19 diagnosis without PCR-confirmed laboratory virus detection (ICD-10-GM: U07.2!) only in order to avoid distortions due to misclassification. We further excluded individuals who were not continuously insured with the respective health insurance organization between January 1, 2019/birth and December 31, 2020/death because relevant outcomes and preexisting health conditions may not have been documented in our data. This exclusion applied to individuals who changed their health insurance organization or emigrated during the observation period.

To minimize differences between COVID-19 and control cohort in terms of covariates that may confound relationships between outcomes and exposure, we used a matching approach (see paragraph “Statistical analyses”). The date of the COVID-19 diagnosis among individuals with COVID-19 was used as the index date for individuals in the COVID-19 cohort as well as for matched controls. Assigning identical index dates to matched COVID-19 and control persons offers the advantages that potential follow-up times are identical and that the timing of nonpharmaceutical interventions (e.g., lockdowns) is equally captured in both cohorts.

Given these matched data, we excluded individuals from the COVID-19 cohort and matched control cases with an index date later than June 30, 2020, as these individuals could not be observed in the post-COVID-19 phase. For the same reason, we excluded individuals who died before reaching the post-COVID-19 phase (see Fig 1).

When analyzing specific health outcomes (see paragraph “Health outcomes”), we further excluded individuals from the analysis of post-COVID-19 incidence if the considered outcome was documented at least once within a 1-year look-back period (i.e., within the 4 quarters preceding the index date). To maintain balance of cohorts regarding covariates, we excluded a complete matched group of COVID-19 and control persons if the outcome was preexisting for the individual with COVID-19 or all of his/her matched control persons. For estimation, we weighted data from individuals in the control cohort with the inverse number of persons remaining in the respective matched group (i.e., weights between 1/5 and 1) to ensure that total weights in the control cohort add up to the number of persons in the COVID-19 cohort.

### Health outcomes

To account for the heterogeneity of potential long-term sequelae of COVID-19 highlighted in the literature [2–4,6–8,39], we selected a large set of outcomes covering multiple organ systems and diagnosis/symptom complexes. Based on published literature, previous work on core outcome set development [16], and clinical expertise in the author team, we selected 96 individual health outcomes potentially related to post-COVID-19. These outcomes constitute new-onset morbidity documented by a physician or psychotherapist within the statutory healthcare system. Operationalization of these outcomes was based on inpatient and outpatient diagnoses according to the ICD-10-GM coding system and the guidelines for good practice secondary data analysis (GPS) of the German Society for Epidemiology (DGEpi) [40]. We aggregated the 96 individual health outcomes into 13 diagnosis/symptom complexes and 3 outcome domains (physical health, mental health, and physical/mental overlap domain). An overview of the outcomes and their grouping is provided in the Supporting information (Section C in S1 Appendix). Aggregation of outcomes into groups offers the advantage of higher statistical power, particularly in the case of post-COVID-19, which is considered to include multiple rare symptoms and diagnoses [6,7,38]. However, aggregation may also obscure relevant heterogeneity in outcomes. Hence, our analysis considered both individual health outcomes and aggregate health outcome groups.

### Covariates

For each individual, we used information on preexisting medical conditions in the 4 quarters preceding the index date. We selected preexisting medical conditions potentially confounding the association between exposure (COVID-19) and incident health outcomes based on published evidence and clinical expertise. These included 13 preexisting medical conditions for children/adolescents and 38 preexisting medical conditions for adults (see Section D in S1 Appendix). In line with previous studies [5,6], we also considered age and sex as well as the severity of COVID-19 as a covariate with potential influence on post-COVID-19 by distinguishing between (1) individuals with outpatient diagnoses of COVID-19 only; (2) individuals with at least 1 hospital visit with COVID-19 diagnosis; and (3) individuals with intensive care and/or ventilation (ICU) with COVID-19 diagnosis.

### Statistical analyses

To select the control cohort, we applied 1:5-matching with replacement [41]. For each individual in the COVID-19 cohort, we selected 5 control persons with identical age (in years) and sex. We chose exact matching on these characteristics to facilitate stratified analysis, e.g., for different age groups. In addition, we accounted for the presence of the medical conditions (described in the section “Covariates”) by propensity score matching. Given different sets of preexisting medical conditions considered as covariates, we estimated separate regression models for children/adolescents and adults. Estimation of the propensity score was based on logistic regression. No caliper was used for matching based on the estimated propensity scores.

Based on these matched data, we estimated differences between COVID-19 and control cohort regarding incidence rates (IRs) of outcomes per 1,000 person-years using Poisson regression [42]. When the conditional mean function is correctly specified, Poisson regression yields consistent estimators of model coefficients irrespective of the distribution of the outcome [42]. Hence, Poisson regression may also be used for binary outcomes as considered in our analysis [43]. Utilizing a main advantage of Poisson regression, we adjusted for differences in individual-specific times at risk (time between index date and end of observation period or death) due to inclusion of these times as offset in the model. In addition, Poisson regression offers the opportunity to adjust for additional covariates that were not considered in the matching process. Based on the results of Poisson regressions, we derived incidence rate ratios (IRRs) with 95% confidence intervals (95% CIs) to characterize relative incidence in COVID-19 and control cohort. We derived *p*-values for estimated IRRs using Z-tests.

To assess the plausibility and robustness of our results, we conducted additional sensitivity analyses. First, we estimated IRRs for all health outcomes documented in the same quarter as the index date or later. In this analysis, we included all individuals with COVID-19 in 2020 and their matched controls. Differences in estimated IRRs between this sample and the post-COVID-19 sample used in the main analysis may indicate possible changes in health differentials between individuals with and without COVID-19 over time. Second, we defined variables capturing hospitalizations (0 = no hospitalization, 1 = at least 1 hospitalization) and the number of quarters with at least 1 physician visit (0,1,…,4) within the 4 quarters preceding the index date. We included these variables as categorical covariates (i.e., in the form of dummy variables) in Poisson regressions to adjust for differences in healthcare utilization between the COVID-19 and the control cohort prior to the index date. If such differences were not fully mitigated by matching, adjustment for hospitalizations and physician visits prior to the index date may yield qualitatively different IRR estimates.

We conducted all statistical analyses using R (version 3.6.1) [44].

Since pooling of individual-level data was not possible due to data protection restrictions, the 6 health insurance datasets were analyzed separately by authorized institutes or the healthcare research department within the respective health insurance organization. To synthesize evidence across datasets, we used the fact that Poisson regression models can be estimated based on individual-level or aggregate data with identical point estimates [45]. Each authorized institute calculated the required aggregate statistics and provided them to ZEGV, where regressions based on combined aggregate data were performed.

## Results

### Descriptive statistics

After excluding individuals without continuous insurance and U07.2! diagnosis only, 5,421,270 children/adolescents and 29,600,265 adults were eligible for inclusion in our study (Fig 1). In 2020, 57,763 of these children/adolescents and 621,205 of these adults, respectively, had a U07.1! diagnosis. In our final sample, 157,134 individuals (11,950 children/adolescents and 145,184 adults) who received their first COVID-19 diagnosis before July 2020 were included. For correspondence with estimation results, data from individuals in the control cohort were weighted according to the inverse size of the respective matched group for descriptive analysis (Table 1; the full descriptive statistics are provided in Section E in S1 Appendix). The distributions of covariates in the COVID-19 and the matched control cohort were similar for both children/adolescents and adults, which indicated successful balancing. Successful balancing was also reflected in almost identical distributions of the propensity scores in both cohorts (see Section E in S1 Appendix). The prevalence of medical conditions selected as covariates was generally lower in children/adolescents than in adults. While our sample included 8,407 (5.8%) hospitalized adults and 3,075 (2.1%) adults with intensive care and/or ventilation, smaller proportions of included children/adolescents were hospitalized with COVID-19 (*n* = 117; 1.0%) and received intensive care and/or ventilation (*n* = 51; 0.4%). The average follow-up time since index date was 236 days (standard deviation (SD) = 44 days, range = 121 to 339 days) in children/adolescents and 254 days (SD = 36 days, range = 93 to 340 days) in adults. Descriptive statistics for all individuals with COVID-19 diagnosis in 2020 (including those with diagnosis in July 2020 or later), who were included in a sensitivity analysis, are shown in the Supporting information (Section F in S1 Appendix).

**Table 1:** Characteristics of COVID-19 and control cohort after matching.

Variable	Category	n COVID-19	Percent COVID-19	Sum of weights Control	Percent Control
Children/adolescents		11,950	100%	11,950.0	100%
Age	0–11	8,032	67.2%	8,032.0	67.2%
	12–17	3,918	32.8%	3,918.0	32.8%
Sex	Female	5,745	48.1%	5,745.0	48.1%
	Male	6,205	51.9%	6,205.0	51.9%
Severity of COVID-19	Outpatient	11,782	98.6%		
	Hospital	117	1.0%		
	ICU	51	0.4%		
Adults		145,184	100%	145,184.0	100%
Age	18–24	12,815	8.8%	12,815.0	8.8%
	25–39	36,565	25.2%	36,565.0	25.2%
	40–49	24,823	17.1%	24,823.0	17.1%
	50–54	16,291	11.2%	16,291.0	11.2%
	55–59	16,332	11.2%	16,332.0	11.2%
	60–64	11,599	8.0%	11,599.0	8.0%
	65–69	6,035	4.2%	6,035.0	4.2%
	70–74	4,700	3.2%	4,700.0	3.2%
	75–79	4,586	3.2%	4,586.0	3.2%
	80-plus	11,438	7.9%	11,438.0	7.9%
Sex	Female	87,395	60.2%	87,395.0	60.2%
	Male	57,789	39.8%	57,789.0	39.8%
Severity of COVID-19	Outpatient	133,702	92.1%		
	Hospital	8,407	5.8%		
	ICU	3,075	2.1%		

Depending on the number of matched controls per individual with COVID-19, each control case enters the analysis with a weight between 1/5 and 1; based on these weights, the column “Sum of weights” represents the sum of the weighted frequencies.

### Incidence of documented health outcomes

To identify the most frequently documented long-term health problems among persons with COVID-19, we considered outcomes with an incidence of at least 1/100 person-years in the COVID-19 cohort. We then sorted these outcomes by IRR for children/adolescents (Table 2) and adults (Table 3). The resulting lists had 5 identical outcomes (cough, fever, headache, malaise/fatigue/exhaustion, and throat/chest pain) across age groups. The outcomes with the highest IRRs in children and adolescents were malaise/fatigue/exhaustion (IRR: 2.28, 95% CI: 1.71 to 3.06, *p* < 0.01, IR COVID-19: 12.58, IR Control: 5.51), cough (IRR: 1.74, 95% CI: 1.48 to 2.04, *p* < 0.01, IR COVID-19: 36.56, IR Control: 21.06), and throat/chest pain (IRR: 1.72, 95% CI: 1.39 to 2.12, *p* < 0.01, IR COVID-19: 20.01, IR Control: 11.66). The outcomes with the largest IRRs in adults were disturbances of smell and taste (IRR: 6.69, 95% CI: 5.88 to 7.60, *p* < 0.01, IR COVID-19: 12.42, IR Control: 1.86), fever (IRR: 3.33, 95% CI: 3.01 to 3.68, *p* < 0.01, IR COVID-19: 11.53, IR Control: 3.46), and dyspnea (IRR: 2.88, 95% CI: 2.74 to 3.02, *p* < 0.01, IR COVID-19: 43.91, IR Control: 15.27). IRRs in adults were higher than in children/adolescents for most outcomes assessed. For all listed outcomes, estimated IRRs were statistically significant. While the unspecific diagnosis malaise/fatigue/exhaustion (ICD-10-GM: R53) was represented in the lists for both children/adolescents and adults, the chronic fatigue syndrome (ICD-10-GM: G93.3) was not. However, chronic fatigue syndrome was also coded more frequently in the COVID-19 than in the control cohort in adults (IRR: 3.04, 95% CI: 2.66 to 3.48, *p* < 0.01, IR COVID-19: 5.94, IR Control: 1.95). In children, the estimated IRR was greater than 1 but not statistically significant (IRR: 1.25, 95% CI: 0.24 to 6.65, *p* = 0.79, IR COVID-19: 0.26, IR Control: 0.21). Estimation results for all health outcomes are shown in the Supporting information (Section G in S1 Appendix).

**Table 2:** Ten post-COVID-19 outcomes in children/adolescents with highest IRRs and incidence of at least 1/100 person-years in the COVID-19 cohort.

Rank	Name	IRR	95% CI	*p*	IR COVID-19	IR Control
1	Malaise/fatigue/exhaustion	2.28	(1.71–3.06)	<0.01	12.58	5.51
2	Cough	1.74	(1.48–2.04)	<0.01	36.56	21.06
3	Throat/chest pain	1.72	(1.39–2.12)	<0.01	20.01	11.66
4	Adjustment disorder	1.71	(1.42–2.06)	<0.01	26.37	15.40
5	Somatization disorder	1.62	(1.30–2.02)	<0.01	17.90	11.06
6	Headache	1.58	(1.35–1.84)	<0.01	36.67	23.24
7	Fever	1.56	(1.30–1.87)	<0.01	27.84	17.84
8	Anxiety disorder	1.54	(1.23–1.92)	<0.01	16.70	10.87
9	Abdominal pain	1.45	(1.27–1.64)	<0.01	53.94	37.31
10	Depression	1.45	(1.12–1.87)	<0.01	12.05	8.32

IR, incidence rate per 1,000 person-years; IRR, incidence rate ratio; 95% CI, 95% confidence interval; *p*-values are derived from Z-tests of IRRs.

**Table 3:** Ten post-COVID-19 outcomes in adults with highest IRRs and incidence of at least 1/100 person-years in the COVID-19 cohort.

Rank	Name	IRR	95% CI	*p*	IR COVID-19	IR Control
1	Disturbances of smell and taste	6.69	(5.88–7.60)	<0.01	12.42	1.86
2	Fever	3.33	(3.01–3.68)	<0.01	11.53	3.46
3	Dyspnea	2.88	(2.74–3.02)	<0.01	43.91	15.27
4	Cough	2.80	(2.64–2.97)	<0.01	29.95	10.71
5	Respiratory insufficiency	2.47	(2.28–2.69)	<0.01	13.76	5.56
6	Throat/chest pain	2.20	(2.09–2.31)	<0.01	34.57	15.74
7	Hair loss	2.02	(1.88–2.18)	<0.01	13.96	6.90
8	Malaise/fatigue/exhaustion	1.97	(1.89–2.06)	<0.01	42.91	21.74
9	Dysphagia	1.95	(1.78–2.12)	<0.01	10.55	5.42
10	Headache	1.74	(1.67–1.82)	<0.01	40.48	23.24

IR, incidence rate per 1,000 person-years; IRR, incidence rate ratio; 95% CI, 95% confidence interval; *p*-values are derived from Z-tests of IRRs.

### Incidence of documented health outcome groups

**Fig 2:**
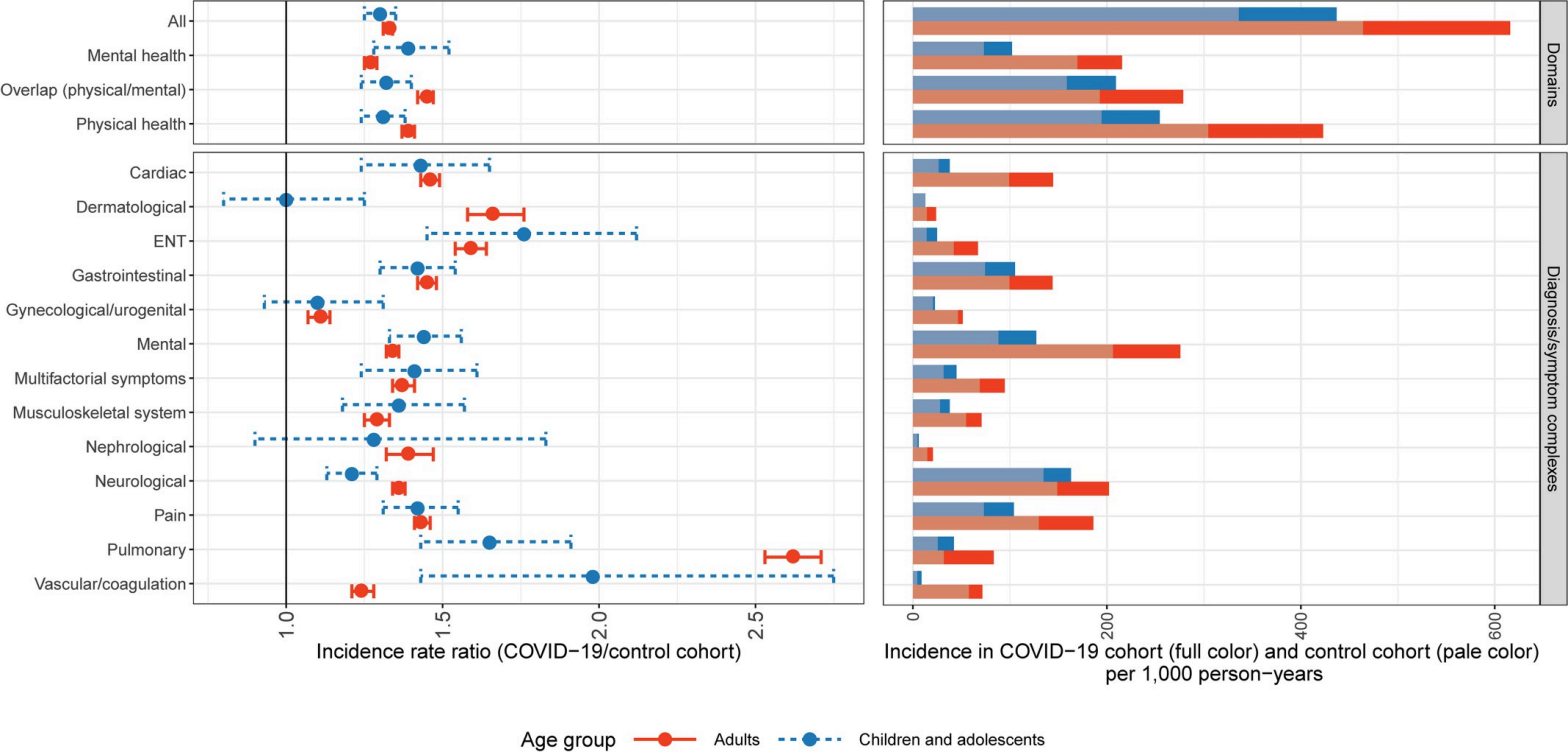
Estimated IRRs with 95% CIs and IRs per 1,000 person-years in COVID-19 cohort for children/adolescents and adults by outcome domain and diagnosis/symptom complex. IRs in the control cohort are shown in pale color. COVID-19, Coronavirus Disease 2019; ENT, ear, nose and throat; IR, incidence rate; IRR, incidence rate ratio; 95% CI, 95% confidence interval.

Considering all outcomes combined, the IR per 1,000 person-years of documented health problems in the COVID-19 cohort was significantly higher than that in the control cohort (upper left panel of Fig 2). This finding holds for both children/adolescents (IRR: 1.30, 95% CI: 1.25 to 1.35, *p* < 0.01, IR COVID-19: 436.91, IR Control: 335.98) and adults (IRR: 1.33, 95% CI: 1.31 to 1.34, *p* < 0.01, IR COVID-19: 615.82, IR Control: 464.15). Furthermore, we found significantly higher IRs in the COVID-19 cohort compared with the control cohort across all considered outcome domains, i.e., physical health (children/adolescents: IRR: 1.31, 95% CI: 1.24 to 1.38, *p* < 0.01, IR COVID-19: 254.58, IR Control: 194.45; adults: IRR: 1.39, 95% CI: 1.37 to 1.41, *p* < 0.01, IR COVID-19: 422.87, IR Control: 304.42), mental health (children/adolescents: IRR: 1.39, 95% CI: 1.28 to 1.52, *p* < 0.01, IR COVID-19: 102.17, IR Control: 73.24; adults: IRR: 1.27, 95% CI: 1.25 to 1.29, *p* < 0.01, IR COVID-19: 215.62, IR Control: 169.50), and the physical/mental overlap domain (children/adolescents: IRR: 1.32, 95% CI: 1.24 to 1.40, *p* < 0.01, IR COVID-19: 209.26, IR Control: 158.71; adults: IRR: 1.45, 95% CI: 1.42 to 1.47, *p* < 0.01, IR COVID-19: 278.58, IR Control: 192.59). For all 13 diagnosis/symptom complexes, IRs in the adult COVID-19 cohort were significantly higher than those in the adult control cohort (lower left panel of Fig 2). In children/adolescents, significantly higher IRs in the COVID-19 cohort compared with the control cohort were observed for 10 out of the 13 defined diagnosis/symptom complexes. Estimated IRRs ranged from 1.00 (95% CI: 0.80 to 1.25, *p* = 1.00; dermatological diagnosis/symptom complex) to 1.98 (95% CI: 1.43 to 2.75, *p* < 0.01; vascular/coagulation diagnosis/symptom complex) in children/adolescents and from 1.11 (95% CI: 1.07 to 1.14, *p* < 0.01; gynecological/urogenital diagnosis/symptom complex) to 2.62 (95% CI: 2.53 to 2.71, *p* < 0.01; pulmonary diagnosis/symptom complex) in adults. Given a 162% higher post-COVID-19 IR in the adult COVID-19 cohort compared with the adult control cohort, pulmonary diagnosis/symptom complex showed the most pronounced overall difference. Stratified estimations for the age groups 0 to 11 and 12 to 17 yielded similar IRRs (see Section H in S1 Appendix).

Across all outcome domains and diagnosis/symptom complexes, IRs in the COVID-19 cohort were lower in children/adolescents than in adults (right panels of Fig 2). Regarding all outcomes combined, the IR in adults with COVID-19 (IR = 615.82) was 41% higher than the IR in children and adolescents with COVID-19 (IR = 436.91).

The full results are presented in the Supporting information (Section I in S1 Appendix).

### Incidence of documented health outcome groups by severity of COVID-19

**Fig 3:**
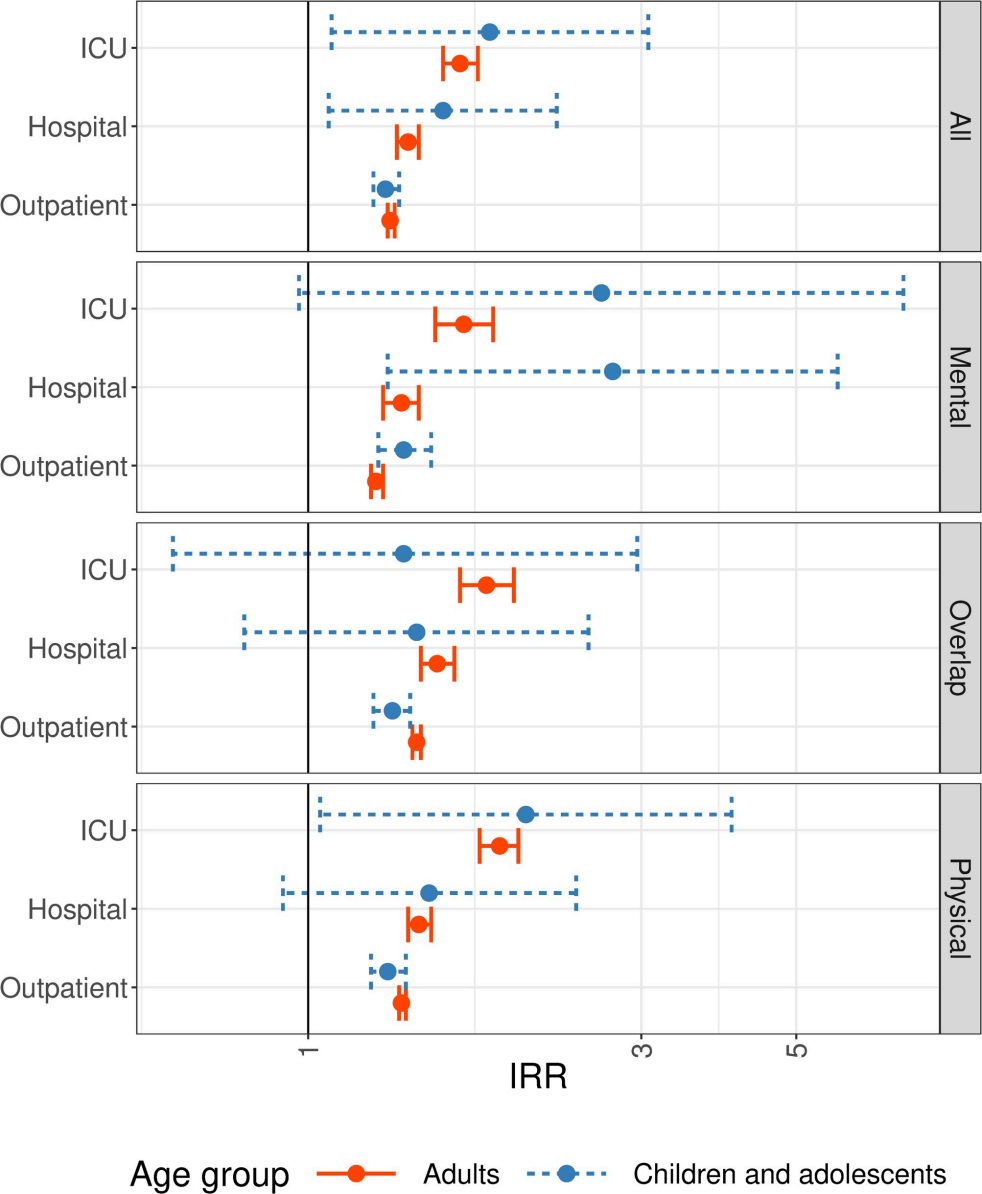
Estimated IRRs with 95% CIs in children/adolescents and adults by severity of COVID-19 and domain. COVID-19, Coronavirus Disease 2019; ICU, intensive care unit; IRR, incidence rate ratio; 95% CI, 95% confidence interval. Estimation results are shown on the log-scale.

Estimations stratified by severity indicated that IRRs for individuals with hospital visits or intensive care were higher than IRRs for individuals with outpatient diagnoses of COVID-19 only (Fig 3). This result holds for both children/adolescents and adults. However, due to the small number of children/adolescents with COVID-19-related hospital visits and intensive care and/or ventilation, precision of the corresponding IRR estimates was low as reflected in large confidence intervals.

### Results of sensitivity analyses

The IRR estimates obtained when considering all health outcomes newly documented in the quarter of the index date or later were generally higher than the IRR estimates obtained from the post-COVID-19 sample used in our main analysis (see Section J in S1 Appendix). This result holds for both children/adolescents and adults and may indicate decreasing health differentials between individuals with and without COVID-19 over time.

Relative to the control cohort, the COVID-19 cohort showed a slightly higher relative frequency of hospitalizations within the 4 quarters preceding the index date and a higher number of quarters with at least 1 physician visit (see section K in S1 Appendix). However, our main results remained qualitatively robust when adjusting for those differences in healthcare utilization preceding the index date (see section K in S1 Appendix).

## Discussion

This large matched cohort study used routine healthcare data to examine the incidence of 96 prespecified diagnoses potentially associated with post-COVID-19 condition among 157,134 individuals (11,950 children/adolescents and 145,184 adults) with PCR-confirmed SARS-CoV-2 infection as compared with matched controls. Based on a minimum follow-up time of 3 months, we observed higher IRs of newly documented diagnoses in the COVID-19 cohort compared with the control cohort among children/adolescents and adults. The relative magnitude of significantly increased newly documented morbidity in association with COVID-19 was similar among adults and children/adolescents. IRs in the COVID-19 and control cohorts were generally lower in children/adolescents than in adults. There were also some differences in the COVID-19-associated morbidity between adults and children/adolescents. Among adults, IRRs were highest for disturbances of smell and taste (IRR: 6.69), fever (IRR: 3.33), and respiratory symptoms such as dyspnea (IRR: 2.88), cough (IRR: 2.80), and respiratory insufficiency (IRR: 2.47), followed by throat and chest pain, hair loss, malaise/fatigue/exhaustion, dysphagia, and headache. Among children, IRRs were highest for malaise, fatigue/exhaustion (IRR: 2.28), cough (IRR: 1.74), throat/chest pain (IRR: 1.72), adjustment disorder (IRR: 1.71), followed by somatization disorder, headache, fever, anxiety disorder, abdominal pain, and depression. With regard to the 13 aggregated symptom/diagnosis complexes, IRs among adults were all significantly higher in the COVID-19 than in the control cohort with IRRs ranging from 1.11 for gynecological/urogenital diagnoses to 2.62 for pulmonary diagnoses. Among children, IRs in the COVID-19 cohort were significantly higher than in the control cohort in 10 out of 13 symptom/diagnosis complexes with IRRs ranging from 1.00 for dermatological diagnoses to 1.98 in the vascular/coagulation symptom/diagnosis complex. Consistent among adults and children/adolescents, there was a clear gradient in IRRs in association with the severity of COVID-19, with the highest IRRs among those individuals that had received intensive care.

The results of our study add to the evidence from a number of previous controlled studies on post-COVID-19-related symptoms, new diagnoses, and healthcare services utilization. In their rapid review last updated on October 29, 2021, Flatby and colleagues identified 9 controlled studies using different data sources, health outcomes, and follow-up times [4]. All of these studies were limited to adults. About half of these studies focused on mental health or neuropsychiatric outcomes. At 6 months of follow-up, these studies observed significant associations between a history of SARS-COV-2 infection and the prevalence of post-traumatic stress disorder, lower cognitive function scores [24], risk of new-onset dementia [25], the prevalence of a variety of mental health conditions [26], and new-onset neurological and psychiatric diseases, including dementia, cerebrovascular events, parkinsonian syndromes, anxiety, mood disorders, illicit drug use disorders, and insomnia [5]. The remainder of earlier studies investigated a wider range of health outcomes considered to be possible long-term sequelae of SARS-CoV-2 infection. Based on electronic healthcare data mainly from the United States, Taquet and colleagues reported hazard ratios between 1.44 and 2.04 for 9 selected health outcomes (e.g., fatigue/malaise, chest/throat pain headache, other pain) among adults previously diagnosed with COVID-19 compared with matched controls previously diagnosed with influenza 3 to 6 months after infection [39]. Using Danish health registry data, Lund and colleagues reported significantly increased rates of overall general practitioner and outpatient hospital visits as well as newly initiated drug prescriptions in association with a history of SARS-CoV-2 infection [27]. Notably, the follow-up period in this previous study covered 2 weeks to 6 months after a positive or, among controls, negative SARS-CoV-2 test result. A large population-based cohort study comparing SARS-CoV-2 infected and noninfected adults in Norway observed that 13 out of 22 preselected symptoms were significantly associated with SARS-CoV-2 infection at 12 months after infection [28]. In line with results from our study, the strongest associations in this previous report were seen for altered smell or taste, shortness of breath, poor memory, fatigue, and chest pain.

More recently, a nationwide study in Norway used a unique database of all adults tested for SARS-CoV-2 between March 1, 2020 to February 1, 2021 and found increased primary healthcare utilization due to respiratory and general/unspecified conditions in association with SARS-CoV-2 infection 2 to 3 months after infection, but not later at 4 to 6 months after infection [19]. Notably, this previous study included only nonhospitalized adults with positive test results, which may explain why increased primary healthcare use in association with prior SARS-CoV-2 infection was limited in time and specialist care use was not increased in relation to SARS-CoV-2 infection at any time during follow-up [19]. Two recently published controlled studies of post-COVID-19 condition among adults support our approach to investigate a wide range of possible health sequelae of SARS-Cov-2 infection as suggested by previous work based on structured expert consensus [16]. Using a retrospective matched cohort design and United Kingdom primary care data, Subramanian and colleagues observed a significant association of SARS-CoV-2 infection with post-COVID-19 condition as defined by WHO criteria, but also 62 individual symptoms, only partly covered by WHO criteria [22]. In an ongoing prospective, population-based matched cohort study conducted in the north of the Netherlands, Ballering and colleagues identified symptoms significantly related to SARS-CoV-2 infection 90 to 150 days after infection based on comparison of symptom profiles between infected persons and matched controls as well as before and after infection within the group of persons with positive test results or clinically diagnosed COVID-19 [29]. These authors found 1 in 8 adults with a history of SARS-CoV-2 infection to experience long-term symptoms including chest pain, difficulties with breathing, muscle pains, loss of taste or smell, tingling extremities, lump in throat, feeling hot and cold alternately, heavy arms or legs, and general tiredness [29].

Our study extends existing evidence on post-COVID-19 syndrome among children and adolescents. We observed relevant post-COVID-19 healthcare utilization and new-onset morbidity patterns documented by physicians in children and adolescents following SARS-CoV-2 infection in a large sample of patients with confirmed COVID-19 compared with a matched control group. Our results contrast with findings from several earlier epidemiological studies among children and adolescents, which did not observe significant group differences between children and adolescents with COVID-19 and controls [17,30–33]. These differences may possibly be due to high dropout rates and/or high risk of selection bias [30], self-reported outcome assessment [30,31], insufficiently long follow-up time to assess post-COVID-19 outcomes [30,32], and low sample size resulting in low statistical power [17,32,33]. Using national SARS-CoV-2 testing data collected in early January 2021 in the UK, Zavala and colleagues reported a slightly higher frequency of any persisting symptoms in association with a positive PCR test result among children aged 2 to 16 years after 1 month of follow-up [34]. Out of a total of 64 individual health symptoms assessed by questionnaire via parent proxy report, 9 symptoms were significantly more prevalent among children with positive PCR test results compared with matched controls. However, observed differences were limited to children with symptomatic SARS-CoV-2 infection [34].

Several large national studies from the UK, Norway, Denmark, and the US have recently contributed further evidence on the health and social impact of long COVID among children and adolescents with conflicting results [20,21,23,35,36,46]. At 3 months of follow-up, the British national matched cohort study of post-COVID-19 condition among children and adolescents 11 to 17 years of age observed small differences between individuals with positive PCR test results compared with controls with regard to individual health symptoms [46]. However, multiple symptoms were significantly more prevalent in in association with SARS-CoV-2 infection. A nationwide register-based study of children and adolescents 0 to 5 and 6 to 17 years of age in Denmark retrospectively assessed (parent proxy report) group differences in the type and duration of symptoms lasting 4 weeks or longer among children and adolescents with and without positive reverse transcription PCR (RT-PCR) test results for SARS-CoV-2 up to March 2021 [35]. These authors found a significant difference in association with a history of SARS-CoV-2 infection only among children 6 to 17 years of age and most symptoms resolved within 5 months [35]. Conducting a before and after register-based study in Norway, Magnusson and colleagues found a relative increase in primary but not specialist healthcare services utilization among children and adolescents with PCR-confirmed SARS-CoV-2 infection between August 2020 and February 2021 compared with control groups with negative test results or not tested [21]. Observed increases in primary healthcare use in association with SARS-CoV-2 infection were short termed up to 3 months following the receipt of test results among children 6 years of age and older, but still present at 3 to 6 months among children 1 to 5 years of age [21].

Similar to our findings, cross-sectional national studies based on Danish national register data demonstrated that a large number of predefined health symptoms lasting more than 2 or 3 months were significantly more frequent among children and adolescents with a history of a positive SARS-CoV-2 test until July 2021 compared with age- and sex-matched controls [20,36]. Symptoms most frequently associated with prior SARS-CoV-2 infection in these previous analyses included a wide variety of health conditions, e.g., mood swings, cough, and stomach aches among young children 0 to 3 years of age, fatigue, and mood swings among older children [36], and sore throat, chest pain, and palpitations among adolescents [20]. This is in line with our observations, although the limited number of children and adolescents in our study precluded further stratification according to age groups and hence age-specific comparisons. Authors from the US Centers of Disease Control and Prevention recently reported significantly increased IRs of potentially severe symptoms and conditions among children and adolescents aged 0 to 17 years in association with confirmed SARS-CoV-2 infection at follow-up times between 60 and 365 days after testing [23]. Symptoms and conditions associated with COVID-19 included smell and taste disturbances, malaise and fatigue, and musculoskeletal pain, but also severe complications, such as acute pulmonary embolism, myocarditis and cardiomyopathy, type 1 diabetes, and type 2 diabetes. As pointed out by the authors, this matched cohort study was subject to a number of potential sources of bias and needs further confirmation.

In line with previous studies, we observed that IRs in children/adolescents with COVID-19 were generally lower than those in adults. Given similar relative magnitudes of post-COVID-19 outcome incidence, the estimated long-term sequelae of COVID-19 therefore appear to be less pronounced in children and adolescents in absolute terms, but high infection rates emphasize that post-COVID-19 cannot be dismissed among children and adolescents.

The main strength of our analysis is its broad database including more than 150,000 individuals with available data in the post-COVID-19 phase. This unselected sample from all over Germany covers both outpatient and inpatient care and, thus, constitutes a unique and comprehensive source of evidence. The 96 outcomes considered in this study were selected based on published evidence and clinical expertise and provided a sound basis for investigation of potential long-term sequelae of COVID-19 across multiple diagnosis/symptom complexes. Our analysis is based on documented, confirmed diagnoses made by physicians and psychotherapists. Accordingly, our results are not subject to possible distortions resulting from selective, incomplete, or inadequate self-reporting of symptoms but instead rely on information provided by medical professionals. To avoid confounding of the relationships between outcomes and exposure, we applied matching on relevant covariates, i.e., age, sex, and several preexisting medical conditions. The resulting distributions of covariates in the COVID-19 and control cohorts were similar, which indicated successful balancing. Our main findings remained stable when we additionally adjusted IRR estimates for healthcare utilization in the 4 quarters preceding the index date. Therefore, the results of this sensitivity analysis further underline the robustness of our findings. Overall, our results for adults are in accordance with those of previous, international studies based on routine health data [5,6,18,39]. This similarity suggests that external validity is high and provides indirect support for the validity of our findings for children and adolescents. Data preparation and analysis in accordance with the GPS of the DGEpi additionally supports the validity and reliability of our results.

Due to the observational nature of our study, a main limitation is that its design does not induce a causal interpretation of results. This limitation is inherent to all observational studies and, thus, virtually all studies on post-COVID-19. We cannot exclude that our results may be affected by unmeasured confounding, although we minimized differences between COVID-19 and control cohort via matching. Our results may also be subject to detection bias that may arise if the health status of individuals after onset of COVID-19 was more closely monitored and better documented by physicians. Although media coverage of long COVID was less extensive during the observation period than today, some individuals with COVID-19 may have visited physicians because they were concerned of possible long-term sequelae. Physicians, in turn, may have scheduled more follow-up appointments with these patients, which may have resulted in better documentation of morbidity in the COVID-19 cohort compared with the control cohort. Generally, psychological and societal factors like media coverage may have influenced the behavior of individuals included in our study cohorts differently and, thus, caused bias in our IRR estimators. Such psychological and societal factors may also have influenced the types of symptoms and diseases documented by physicians, possibly with a bias toward psychiatric conditions. Individuals with mild or asymptomatic course of COVID-19 are likely to be underrepresented in our study because SARS-CoV-2 infections may not have been documented [47], especially in the first months of the pandemic. The resulting selection of more severe COVID-19 cases may lead to higher incidence estimates in this cohort. By the same token, individuals with undocumented SARS-CoV-2 infection may have been included in the control cohort. To the extent that post-COVID-19 also occurred in persons with undocumented infections, this misclassification induces an overestimation of IRs in the control group and, thus, a bias toward the null in estimators of IRRs. This may particularly be a concern in the analyses of children/adolescents, as acute COVID-19 symptoms are more frequently mild and/or absent in this group so that they may not have resulted in a clinical consultation and thus not have been documented in the data used for this study.

To synthesize evidence across the datasets provided by 6 health insurance organizations, we used the fact that Poisson regression models can be estimated based on aggregate data. While point estimates are identical, variance estimates based on aggregate data tend to be larger than those based on individual-level data. Statistical efficiency gains due to 1:5-matching could not be exploited because of data aggregation. These aspects imply that our results are conservative in terms of statistical significance. A potential underestimation of variance arises from the missing possibility to adjust standard error estimators based on aggregate data to account for multiple representations of the same individuals due to matching with replacement. As a result, the net effect of using aggregate data on variance estimators of IRRs is ambiguous. Against that background, estimates of confidence intervals and *p*-values reported in our study should be interpreted with some caution.

Generally, we assume that diagnoses made by physicians and psychotherapists have higher validity than self-reported outcomes. However, this may not be true for all coded diagnoses, since the use of certain diagnoses might be relevant for billing purposes. In our study, the validity of U07.1! diagnoses is crucial for correct identification of individuals with PCR-confirmed SARS-CoV-2 infection. We cannot fully exclude that some of these diagnoses were documented without PCR confirmation of SARS-CoV-2 infection. Follow-up times after COVID-19 diagnosis in our sample were limited because data were available up to the end of 2020 only. Accordingly, follow-up beyond 3 to 6 months within the post-COVID-19 phase was not possible. While we used a matching approach to balance the COVID-19 and control cohorts regarding age, sex, and preexisting medical conditions, our data did not provide information on ethnicity. Therefore, we cannot exclude that the distribution of ethnicity differed between the study cohorts. We also cannot exclude that outcomes or medical conditions used for propensity score matching were not diagnosed and documented by physicians for some individuals. This could have induced bias in our IRR estimators if COVID-19 and control cohorts differed in the frequency of such unobserved diagnoses. Finally, the dynamic of the COVID-19 pandemic during the study period in 2020 was different than it is currently. Accordingly, utilization of health services has changed over time and is not necessarily the same in 2021 to 2022 as it was early in the pandemic. In addition, recent variants of SARS-CoV-2, including Omicron, were not covered by our sample and a potentially mitigating role of vaccination against SARS-CoV-2 regarding long-term sequelae of COVID-19 could not be assessed. Hence, long-term and ongoing observation is required to assess the generalizability of our results to later phases of the pandemic.

In conclusion, the results of the present study indicate that post-COVID-19 cannot be dismissed among children and adolescents. We found that COVID-19 diagnosis was associated with higher long-term demand for healthcare services as reflected in outpatient and inpatient diagnoses of a broad set of outcomes more than 3 months after confirmed SARS-CoV-2 infection. While children and adolescents appear to be less affected than adults, these findings are statistically significant for all age groups. In children and adolescents, the IRR of documented new-onset mental health problems during follow-up was higher compared with adults, whereas the opposite was true for documented health outcomes of the pulmonary diagnosis/symptom complex. Controlled population-based studies with extended follow-up and further in-depth analyses are required to confirm results among children and adolescents and their impact for individuals and health care systems.

## Supporting information

S1 RECORD Checklist RECORD checklist. (PDF)

S1 Appendix Additional information and analyses. (PDF)

S1 File Outcome definitions. ICD-10-GM codes used for operationalization of outcomes.
(CSV)

## Abbreviations

COVID-19: Coronavirus Disease 2019
GPS: good practice secondary data analysis
IR: incidence rate
IRR: incidence rate ratio
PCR: polymerase chain reaction
RT-PCR: reverse transcription PCR
SARS-CoV-2: Severe Acute Respiratory Syndrome Coronavirus 2
SD: standard deviation
WHO: World Health Organization
95% CI: 95% confidence interval
