# Supplementary material for: Post-COVID-19-associated morbidity in children, adolescents, and adults: A matched cohort study including more than 157,000 individuals with COVID-19 in Germany
Source: PLoS Med. 2022 Nov 10;19(11):e1004122. doi: 10.1371/journal.pmed.1004122 (PMC9648706; doi:10.1371/journal.pmed.1004122)
Supplement: S1 Appendix — (PDF) [file pmed.1004122.s002.pdf]

S2 Appendix: Additional information and analyses

Post COVID-19 associated morbidity in children, adolescents, and adults: A matched cohort study including more than 157,000 individuals with COVID-19 in Germany

Martin Roessler[1], Falko Tesch[1], Manuel Batram[2], Josephine Jacob[3], Friedrich Loser[4], Oliver Weidinger[5], Danny Wende[6], Annika Vivirito[3], Nicole Toepfner[7], Franz Ehm[1], Martin Seifert[1], Oliver Nagel[3], Christina König[4], Roland Jucknewitz[5], Jakob Peter Armann[7], Reinhard Berner[7], Marina Treskova-Schwarzbach[8], Dagmar Hertle[6], Stefan Scholz[8], Stefan Stern[5], Pedro Ballesteros[6], Stefan Baßler[9], Barbara Bertele[4], Uwe Repschläger[6], Nico Richter[10], Cordula Riederer[10], Franziska Sobik[10], Anja Schramm[5], Claudia Schulte[6], Lothar Wieler[8], Jochen Walker[3], Christa Scheidt-Nave[8], Jochen Schmitt[1]

[1]Center for Evidence-Based Healthcare (ZEGV), University Hospital Carl Gustav Carus and Carl Gustav Carus Faculty of Medicine, TU Dresden, Dresden, Germany
[2]Vandage GmbH, Bielefeld, Germany and Faculty for Business Administration and Economics, Bielefeld University, Bielefeld, Germany
[3]InGef - Institute for Applied Health Research Berlin, Berlin, Germany
[4]Techniker Krankenkasse, Hamburg, Germany
[5]AOK Bayern - Die Gesundheitskasse, Regensburg, Germany
[6]BARMER Institut für Gesundheitssystemforschung (bifg), Berlin, Germany
[7]Department of Pediatrics, University Hospital Carl Gustav Carus and Carl Gustav Carus Faculty of Medicine, TU Dresden, Dresden, Germany,
[8]Robert Koch-Institute, Berlin, Germany
[9]AOK PLUS, Dresden, Germany,
[10]DAK-Gesundheit, Hamburg, Germany

Corresponding author: Martin Roessler, Center for Evidence-Based Healthcare (ZEGV), University Hospital Carl Gustav Carus and Carl Gustav Carus Faculty of Medicine, TU Dresden, Fetscherstr. 74, 01307 Dresden, Germany. e-mail: martin.roessler@uniklinikum-dresden.de

A) The POINTED program

The Post-COVID-19 Monitoring in Routine Health Insurance Data (POINTED) program was established in December 2020 as part of the German Network University Medicine project egePan Unimed, which focuses on the development, evaluation, and implementation of evidence-based pandemic management. The overarching aim of the POINTED program is to rapidly generate new evidence on prioritized questions concerning pandemic response with substantial public health impact by the use of healthcare data. The POINTED consortium is coordinated by the Center for Evidence-Based Healthcare (ZEGV) at the TU Dresden and consists of large German statutory health insurance organizations, health services research institutes (ZEGV and InGef - Institute for Applied Health Research Berlin), the Robert Koch Institute (RKI), and clinical experts.

B) Definition of post COVID-19 phase

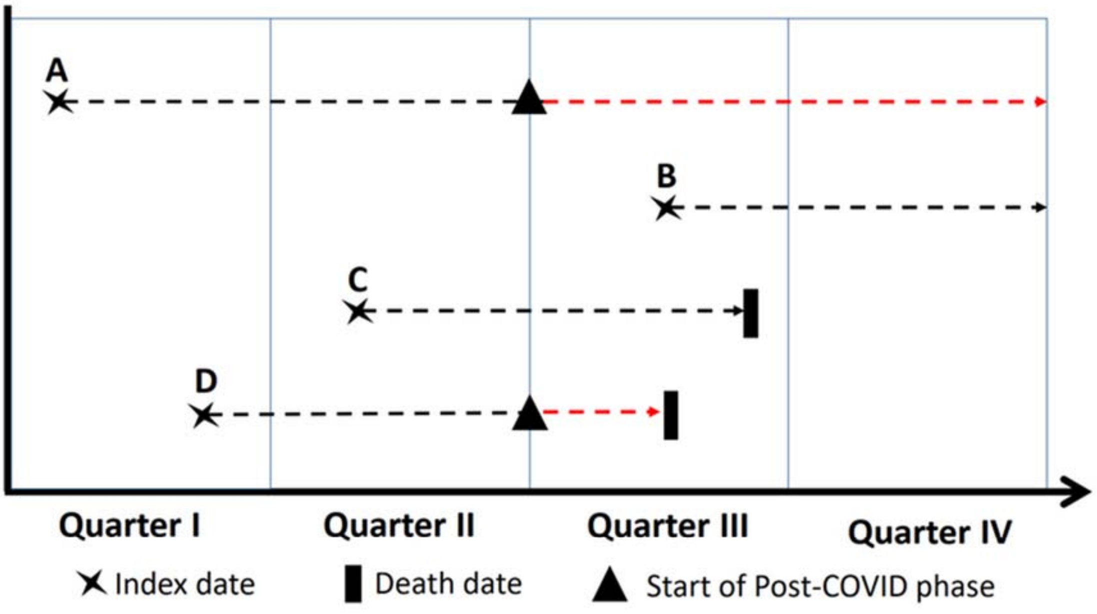

- Person A: All outcomes first observed in quarters III and IV are classified as incident Post-COVID outcomes
- Person B: Person was not observed in second quarter after index date $\Longrightarrow$ not included in POST-COVID analyses
- Person C: Person died before second quarter after index date $\Longrightarrow$ not included in Post-COVID analyses
- Person D: All outcomes first observed between start of quarter III and death are classified as incident Post-COVID outcomes

Fig S1: Definition of post COVID-19 phase

C) Outcomes by domain and diagnosis/symptom complex

Outcomes by domain

Mental health outcomes: Adjustment disorder; Anxiety disorder; Behavioral symptoms; Cognitive function impairment; Concentration impairment/Concentration deficit; Depression; Disorientation; Emotional and behavioral disorder; Mood disorder; Obsessive-compulsive disorder; Somatization disorder

Overlap (physical/mental) outcomes: Abdominal pain; Acute pain; Cachexia; Changes in bowel habits; Chronic fatigue syndrome; Developmental delay; Dysuria; Eye pain; General symptoms; Headache; Hyperhidrosis; Joint pain; Loss of appetite, weight gain/loss, eating disorders; Malaise/fatigue/exhaustion; Memory impairment; Myalgia; Neurasthenia; Other coordination disorders/ataxia; Pain, not elsewhere classified; Paresthesia of skin; Post-COVID; Sensation and perception disorder; Sleep disorders; Somnolence, sopor, coma; Throat/chest pain

Physical health outcomes: Anuria, oliguria; Arthritides; Ascites; Carditis due to viruses; Cough; Covid toe; Diarrhea; Disturbances of smell and taste; Dyslexia; Dysmenorrhea; Dysphagia; Dyspnea; Epistaxis; Facial nerve paralysis; Fever; Flatulence; Gangraen; Hair loss; Hearing loss/tinnitus; Heart failure; Heart murmurs; Heartburn; Hemorrhage; Hepatomegaly and splenomegaly; Hoarseness; Hypotension; Impaired balance; Lymphadenopathy; Meningismus; Movement disorders; Multisystemic inflammatory syndrome; Myocardial infarction; Myocarditis; Nausea; Neurological manifestation of Post-COVID; Oedema; Other cardiac arrhythmias; Other symptoms of the urinary system; Paresis; Pathological findings from male genital tract; Pathological lung findings; Pathological reflexes; Pericarditis; Polyuria; Pulmonary embolism; Rash; Respiratory insufficiency; Seizures; Shock; Sinus vein thrombosis; Speech and language disorders; Stroke; Subcutaneous nodules; Syncope; Tachycardia/Palpitation; Tetany; Thrombosis; Urethral discharge; Urinary retention; Vertigo; Visual disturbances

Outcomes by diagnosis/symptom complex

Cardiac diagnosis/symptom complex: Carditis due to viruses; Heart failure; Heart murmurs; Hypotension; Myocardial infarction; Myocarditis; Other cardiac arrhythmias; Pericarditis; Shock; Syncope; Tachycardia/Palpitation; Throat/chest pain

Dermatological diagnosis/symptom complex: Hair loss; Rash; Subcutaneous nodules

ENT diagnosis/symptom complex: Disturbances of smell and taste; Dysphagia; Epistaxis; Hearing loss/tinnitus; Hoarseness; Vertigo

Gastrointestinal diagnosis/symptom complex: Abdominal pain; Ascites; Changes in bowel habits; Diarrhea; Dysphagia; Flatulence; Heartburn; Hepatomegaly and splenomegaly; Nausea

Gynecological/urogenital diagnosis/symptom complex: Dysmenorrhea; Pathological findings from male genital tract; Urethral discharge

Mental diagnosis/symptom complex: Adjustment disorder; Anxiety disorder; Behavioral symptoms; Chronic fatigue syndrome; Cognitive function impairment; Concentration impairment/Concentration deficit; Depression; Disorientation; Emotional and behavioral disorder; Malaise/fatigue/exhaustion; Memory impairment; Mood disorder; Neurasthenia; Obsessive-compulsive disorder; Other coordination disorders/ataxia; Paresthesia of skin; Sensation and perception disorder; Sleep disorders; Somatization disorder; Somnolence, sopor, coma

Multifactorial symptoms: Acute pain; Cachexia; Fever; General symptoms; Hyperhidrosis; Loss of appetite, weight gain/loss, eating disorders; Lymphadenopathy; Oedema; Pain, not elsewhere classified

Musculoskeletal system diagnosis/symptom complex: Arthritides; Impaired balance; Joint pain; Movement disorders; Myalgia

Nephrological diagnosis/symptom complex: Anuria, oliguria; Dysuria; Other symptoms of the urinary system; Polyuria; Urinary retention

Neurological diagnosis/symptom complex: Chronic fatigue syndrome; Developmental delay; Disturbances of smell and taste; Dyslexia; Dysphagia; Facial nerve paralysis; Impaired balance; Meningismus; Movement disorders; Neurasthenia; Neurological manifestation of Post-COVID; Other

coordination disorders/ataxia; Paresis; Paresthesia of skin; Pathological reflexes; Seizures; Sensation and perception disorder; Sinus vein thrombosis; Sleep disorders; Somnolence, sopor, coma; Speech and language disorders; Stroke; Tetany; Urinary retention; Vertigo; Visual disturbances

Pain diagnosis/symptom complex: Abdominal pain; Acute pain; Dysuria; Eye pain; Headache; Joint pain; Myalgia; Pain, not elsewhere classified; Throat/chest pain

Pulmonary diagnosis/symptom complex: Cough; Dyspnea; Pathological lung findings; Pulmonary embolism; Respiratory insufficiency

Vascular/coagulation diagnosis/symptom complex: Covid toe; Epistaxis; Gangraen; Hemorrhage; Myocardial infarction; Pulmonary embolism; Sinus vein thrombosis; Stroke; Thrombosis

D) Covariates used for propensity score matching

Covariates for children and adolescents

Asthma; Bronchopulmonary dysplasia; Cancer; Congenital heart disease; Diabetes: type I with insulin; Dialysis; Down syndrome; Epilepsy; Immunosuppressive disease; Immunosuppressive therapy; Obesity; Primary immunodeficiency; Psychomotor deficit

Covariates for adults

Asthma; Autoimmune disease; Cardiac arrhythmia; Cerebrovascular disease; Chronic kidney disease; COPD or severe lung disease; Coronary heart disease; Crohn's disease; Dementia; Depression; Diabetes (without insulin); Diabetes: type I with insulin; Diabetes: other with insulin; Dialysis; Down syndrome; Heart failure; Hemato-oncological disease with therapy; Hemato-oncological disease without therapy; Hepatitis; HIV; Hypertension; Immunosuppressive disease; Immunosuppressive therapy; Intellectual disabilities; Interstitial lung disease; Mental illness; Metastatic cancer with therapy; Metastatic cancer without therapy; Obesity; Organ transplant; Other neurological diseases; Primary immunodeficiency; Rheumatological symptoms/diagnosis; Severe lung disease; Severe or cirrhotic liver disease; Solid cancer with therapy; Solid cancer without therapy; Ulcerative colitis

E) Descriptive statistics for COVID-19 and control cohorts

Full descriptive statistics

Table S1: Characteristics of COVID-19 and control cohort after matching

Variable	Category	n COVID-19	Percent COVID-19	Sum of weights Control	Percent Control	Std. diff.
Children/adolescents		11,950	100%	11,950.0	100%	
Age	0-11	8,032	67.2%	8,032.0	67.2%	0.000
	12-17	3,918	32.8%	3,918.0	32.8%	0.000
Sex	female	5,745	48.1%	5,745.0	48.1%	0.000
	male	6,205	51.9%	6,205.0	51.9%	0.000
Asthma		128	1.1%	129.2	1.1%	0.000
Bronchopulmonary dysplasia		7	0.1%	6.6	0.1%	0.000
Cancer		28	0.2%	26.0	0.2%	0.000
Congenital heart disease		253	2.1%	252.0	2.1%	0.000
Diabetes: type I with insulin		34	0.3%	32.2	0.3%	0.000
Dialysis		<5		5.0		
Down syndrome		20	0.2%	16.6	0.1%	0.026
Epilepsy		107	0.9%	104.6	0.9%	0.000
Immunosuppressive disease		113	0.9%	113.6	1.0%	-0.010
Immunosuppressive therapy		33	0.3%	28.0	0.2%	0.020
Obesity		15	0.1%	18.0	0.2%	-0.026
Primary immunodeficiency		62	0.5%	64.2	0.5%	0.000
Psychomotor deficit		381	3.2%	384.8	3.2%	0.000
Severity of COVID-19	outpatient	11,782	98.6%			
	hospital	117	1.0%			
	ICU	51	0.4%			
Adults		145,184	100%	145,184.0	100%	
Age	18-24	12,815	8.8%	12,815.0	8.8%	0.000
	25-39	36,565	25.2%	36,565.0	25.2%	0.000
	40-49	24,823	17.1%	24,823.0	17.1%	0.000
	50-54	16,291	11.2%	16,291.0	11.2%	0.000
	55-59	16,332	11.2%	16,332.0	11.2%	0.000
	60-64	11,599	8.0%	11,599.0	8.0%	0.000
	65-69	6,035	4.2%	6,035.0	4.2%	0.000
	70-74	4,700	3.2%	4,700.0	3.2%	0.000
	75-79	4,586	3.2%	4,586.0	3.2%	0.000
	80-plus	11,438	7.9%	11,438.0	7.9%	0.000
Sex	female	87,395	60.2%	87,395.0	60.2%	0.000
	male	57,789	39.8%	57,789.0	39.8%	0.000
Asthma		4,533	3.1%	4,406.2	3.0%	0.006
Autoimmune disease		12,208	8.4%	12,196.2	8.4%	0.000
Cardiac arrhythmia		6,169	4.2%	6,306.0	4.3%	-0.005
Cerebrovascular disease		8,521	5.9%	8,480.0	5.8%	0.004

Chronic kidney disease	8,194	5.6%	8,407.8	5.8%	-0.009
COPD or severe lung disease	4,628	3.2%	4,186.1	2.9%	0.017
Coronary heart disease	7,053	4.9%	7,192.4	5.0%	-0.005
Crohn’s disease	777	0.5%	673.7	0.5%	0.000
Dementia	5,695	3.9%	5,987.1	4.1%	-0.010
Depression	11,613	8.0%	11,427.9	7.9%	0.004
Diabetes (without insulin)	9,135	6.3%	9,346.7	6.4%	-0.004
Diabetes: type I with insulin	1,161	0.8%	1,102.1	0.8%	0.000
Diabetes: other with insulin	4,177	2.9%	4,396.4	3.0%	-0.006
Dialysis	762	0.5%	508.3	0.4%	0.015
Down syndrome	159	0.1%	146.5	0.1%	0.000
Heart failure	6,653	4.6%	6,866.1	4.7%	-0.005
Hemato-oncological disease with therapy	241	0.2%	182.2	0.1%	0.026
Hemato-oncological disease without therapy	594	0.4%	529.1	0.4%	0.000
Hepatitis	428	0.3%	401.4	0.3%	0.000
HIV	273	0.2%	246.7	0.2%	0.000
Hypertension	31,371	21.6%	31,648.5	21.8%	-0.005
Immunosuppressive disease	3,822	2.6%	3,591.4	2.5%	0.006
Immunosuppressive therapy	2,675	1.8%	2,440.5	1.7%	0.008
Intellectual disabilities	986	0.7%	955.9	0.7%	0.000
Interstitial lung disease	301	0.2%	233.8	0.2%	0.000
Mental illness	1,381	1.0%	1,145.9	0.8%	0.021
Metastatic cancer with therapy	432	0.3%	398.5	0.3%	0.000
Metastatic cancer without therapy	395	0.3%	313.8	0.2%	0.020
Obesity	5,306	3.7%	5,413.9	3.7%	0.000
Organ transplant	226	0.2%	167.4	0.1%	0.026
Other neurological diseases	8,329	5.7%	8,222.0	5.7%	0.000
Primary immunodeficiency	418	0.3%	361.4	0.2%	0.020
Rheumatological symptoms/diagnosis	4,994	3.4%	4,797.6	3.3%	0.006
Severe lung disease	223	0.2%	163.2	0.1%	0.026
Severe or cirrhotic liver disease	638	0.4%	521.6	0.4%	0.000
Solid cancer with therapy	1,098	0.8%	988.8	0.7%	0.012

Solid cancer without therapy		5,821	4.0%	5,465.1	3.8%	0.010
Ulcerative colitis		873	0.6%	825.8	0.6%	0.000
Severity of COVID-19	outpatient	133,702	92.1%			
	hospital	8,407	5.8%			
	ICU	3,075	2.1%			

Std. diff. = standardized difference. Depending on the number of matched controls per individual with COVID-19, each control case enters the analysis with a weight between 1/5 and 1; based on these weights, the column "Sum of weights" represents the sum of the weighted frequencies.

Distributions of propensity scores in COVID-19 and control cohort

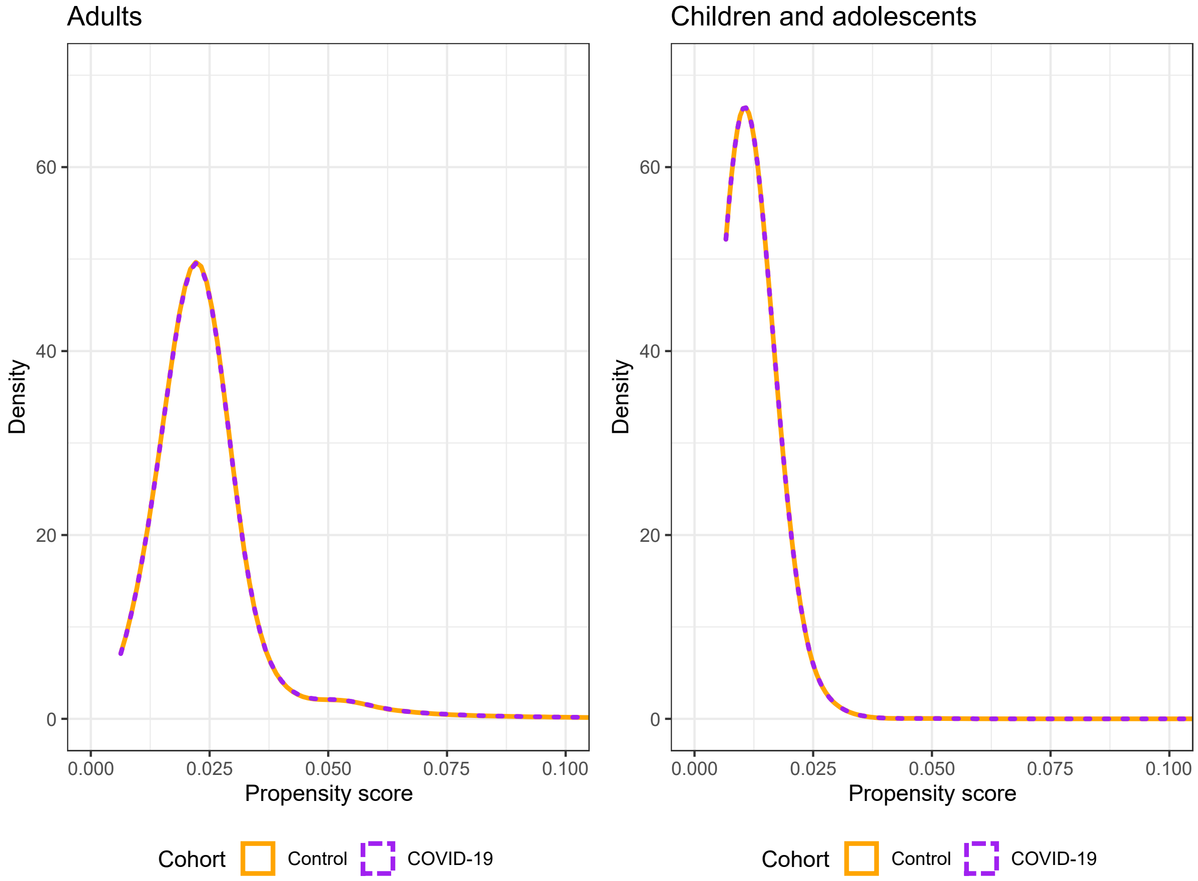

Fig S2: Distributions of propensity scores in COVID-19 and control cohort for children/adolescents and adults

F) Full COVID-19 and Post-COVID sample

Table S2: Characteristics of individuals with COVID-19 included in full COVID-19 and post COVID-19 sample

Variable	Category	n (full COVID-19 sample)	Percent (full COVID-19 sample)	n (post COVID-19 sample)	Percent (post COVID-19 sample)
Children/adolescents		57,763	100%	11,950	100%
Age	00-11	31,345	54.3%	8,032	67.2%
	12-17	26,418	45.7%	3,918	32.8%
Sex	female	28,120	48.7%	5,745	48.1%
	male	29,643	51.3%	6,205	51.9%
Asthma		595	1.0%	128	1.1%
Bronchopulmonary dysplasia		30	0.1%	7	0.1%
Cancer		130	0.2%	28	0.2%
Congenital heart disease		868	1.5%	253	2.1%
Diabetes: type I with insulin		173	0.3%	34	0.3%
Dialysis		9	0.0%	<5	
Down syndrome		70	0.1%	20	0.2%
Epilepsy		501	0.9%	107	0.9%
Immunosuppressive disease		474	0.8%	113	0.9%
Immunosuppressive therapy		110	0.2%	33	0.3%
Obesity		79	0.1%	15	0.1%
Primary immunodeficiency		263	0.5%	62	0.5%
Psychomotor deficit		1,577	2.7%	381	3.2%
Severity of COVID-19	outpatient	56,827	98.4%	11,782	98.6%
	hospital	723	1.3%	117	1.0%
	ICU	213	0.4%	51	0.4%
Adults		621,202	100%	145,184	100%
Age	18-24	60,425	9.7%	12,815	8.8%
	25-39	150,971	24.3%	36,565	25.2%
	40-49	100,624	16.2%	24,823	17.1%
	50-54	63,011	10.1%	16,291	11.2%
	55-59	63,260	10.2%	16,332	11.2%
	60-64	45,132	7.3%	11,599	8.0%
	65-69	25,082	4.0%	6,035	4.2%
	70-74	20,886	3.4%	4,700	3.2%
	75-79	23,047	3.7%	4,586	3.2%
	80-plus	68,764	11.1%	11,438	7.9%
Sex	female	358,777	57.8%	87,395	60.2%
	male	262,425	42.2%	57,789	39.8%
Asthma		17,694	2.8%	4,533	3.1%
Autoimmune disease		50,193	8.1%	12,208	8.4%
Cardiac arrhythmia		35,087	5.6%	6,169	4.2%
Cerebrovascular disease		43,354	7.0%	8,521	5.9%
Chronic kidney disease		48,396	7.8%	8,194	5.6%

COPD or severe lung disease		19,557	3.1%	4,628	3.2%
Coronary heart disease		39,389	6.3%	7,053	4.9%
Crohn's disease		2,865	0.5%	777	0.5%
Dementia		36,104	5.8%	5,695	3.9%
Depression		48,221	7.8%	11,613	8.0%
Diabetes (without insulin)		51,040	8.2%	9,135	6.3%
Diabetes: type I with insulin		5,173	0.8%	1,161	0.8%
Diabetes: other with insulin		23,236	3.7%	4,177	2.9%
Dialysis		3,310	0.5%	762	0.5%
Down syndrome		694	0.1%	159	0.1%
Heart failure		39,589	6.4%	6,653	4.6%
Hemato-oncological disease with therapy		1,068	0.2%	241	0.2%
Hemato-oncological disease without therapy		2,863	0.5%	594	0.4%
Hepatitis		1,860	0.3%	428	0.3%
HIV		914	0.1%	273	0.2%
Hypertension		155,603	25.0%	31,371	21.6%
Immunosuppressive disease		16,621	2.7%	3,822	2.6%
Immunosuppressive therapy		11,483	1.8%	2,675	1.8%
Intellectual disabilities		5,496	0.9%	986	0.7%
Interstitial lung disease		1,406	0.2%	301	0.2%
Mental illness		6,178	1.0%	1,381	1.0%
Metastatic cancer with therapy		2,188	0.4%	432	0.3%
Metastatic cancer without therapy		2,102	0.3%	395	0.3%
Obesity		25,112	4.0%	5,306	3.7%
Organ transplant		955	0.2%	226	0.2%
Other neurological diseases		41,306	6.6%	8,329	5.7%
Primary immunodeficiency		1,516	0.2%	418	0.3%
Rheumatological symptoms/diagnosis		21,464	3.5%	4,994	3.4%
Severe lung disease		946	0.2%	223	0.2%
Severe or cirrhotic liver disease		3,219	0.5%	638	0.4%
Solid cancer with therapy		4,964	0.8%	1,098	0.8%
Solid cancer without therapy		27,348	4.4%	5,821	4.0%
Ulcerative colitis		3,533	0.6%	873	0.6%
Severity of COVID-19	outpatient	548,159	88.2%	133,702	92.1%
	hospital	55,129	8.9%	8,407	5.8%
	ICU	17,914	2.9%	3,075	2.1%

G) Estimation results for all health outcomes

Please note that missing IRRs indicate that incidence was zero in the COVID-19 and/or control cohort.

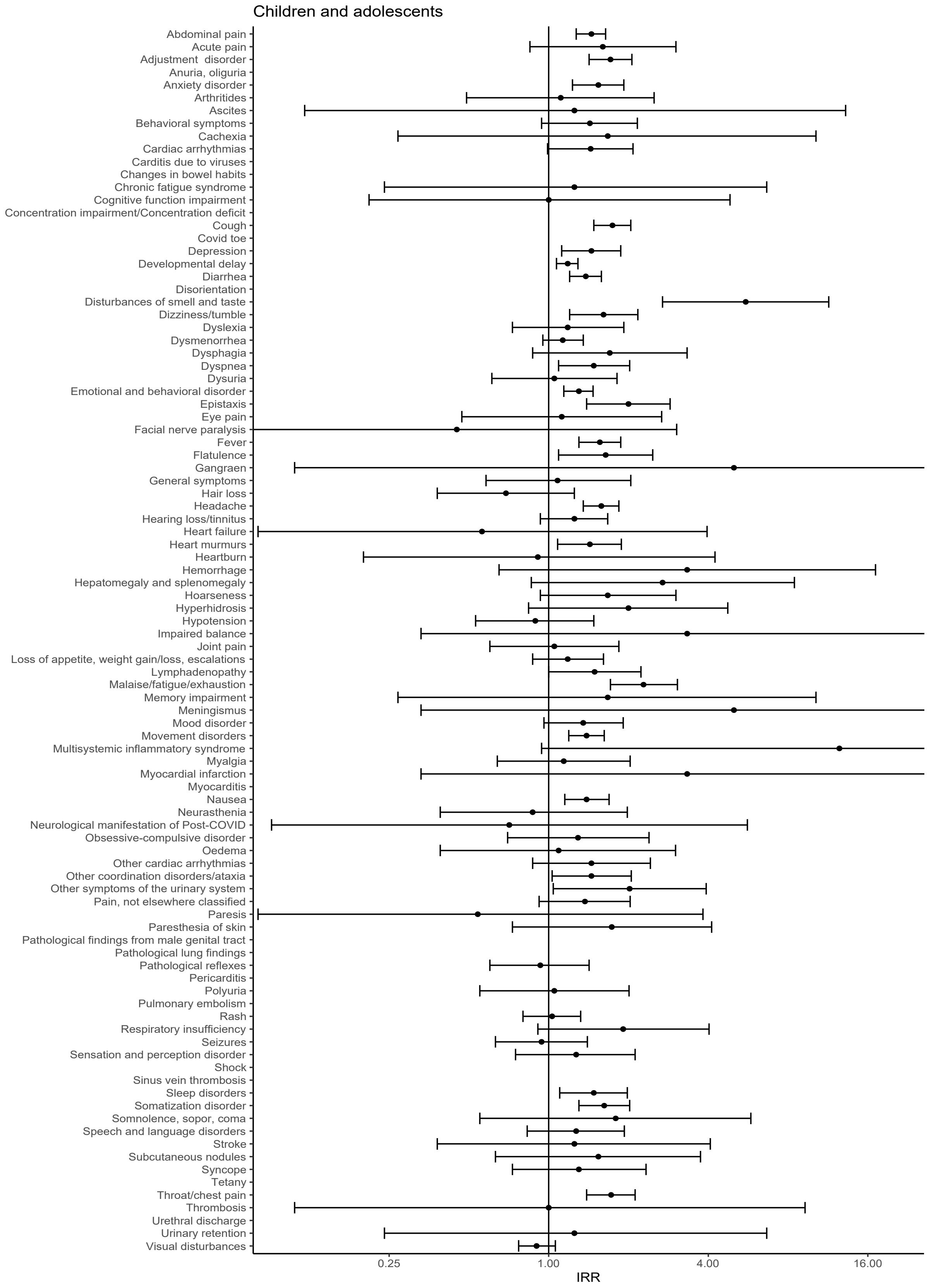

Fig S3: Estimation results for health outcomes in children and adolescents

IRR = incidence rate ratio; estimation results are shown on the log-scale; estimation of IRR was not possible if incidence was zero in the COVID-19 or control cohort.

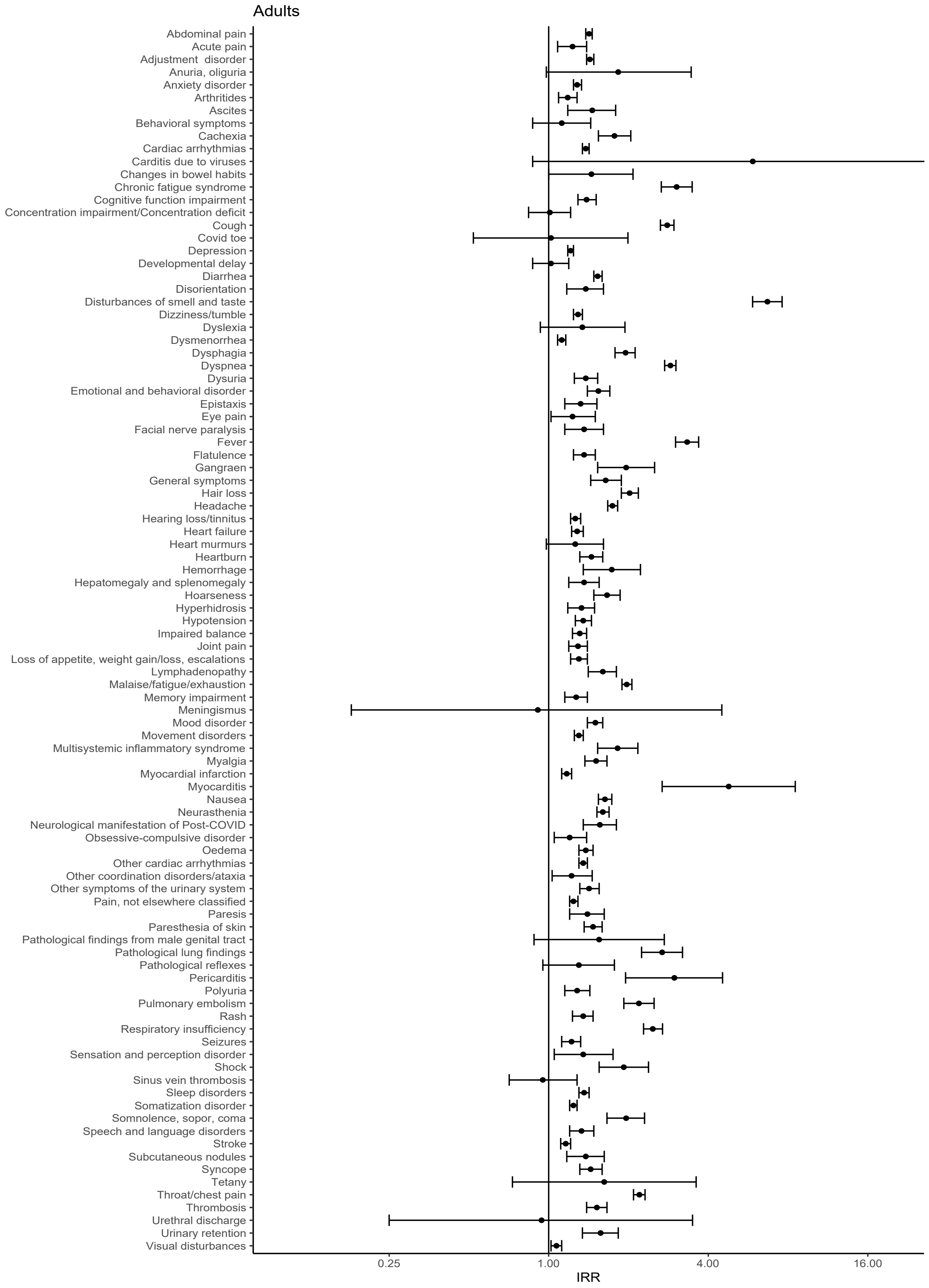

Fig S4: Estimation results for health outcomes in adults

IRR = incidence rate ratio; estimation results are shown on the log-scale; estimation of IRR was not possible if incidence was zero in the COVID-19 or control cohort.

H) Results for age groups 0-11 and 12-17

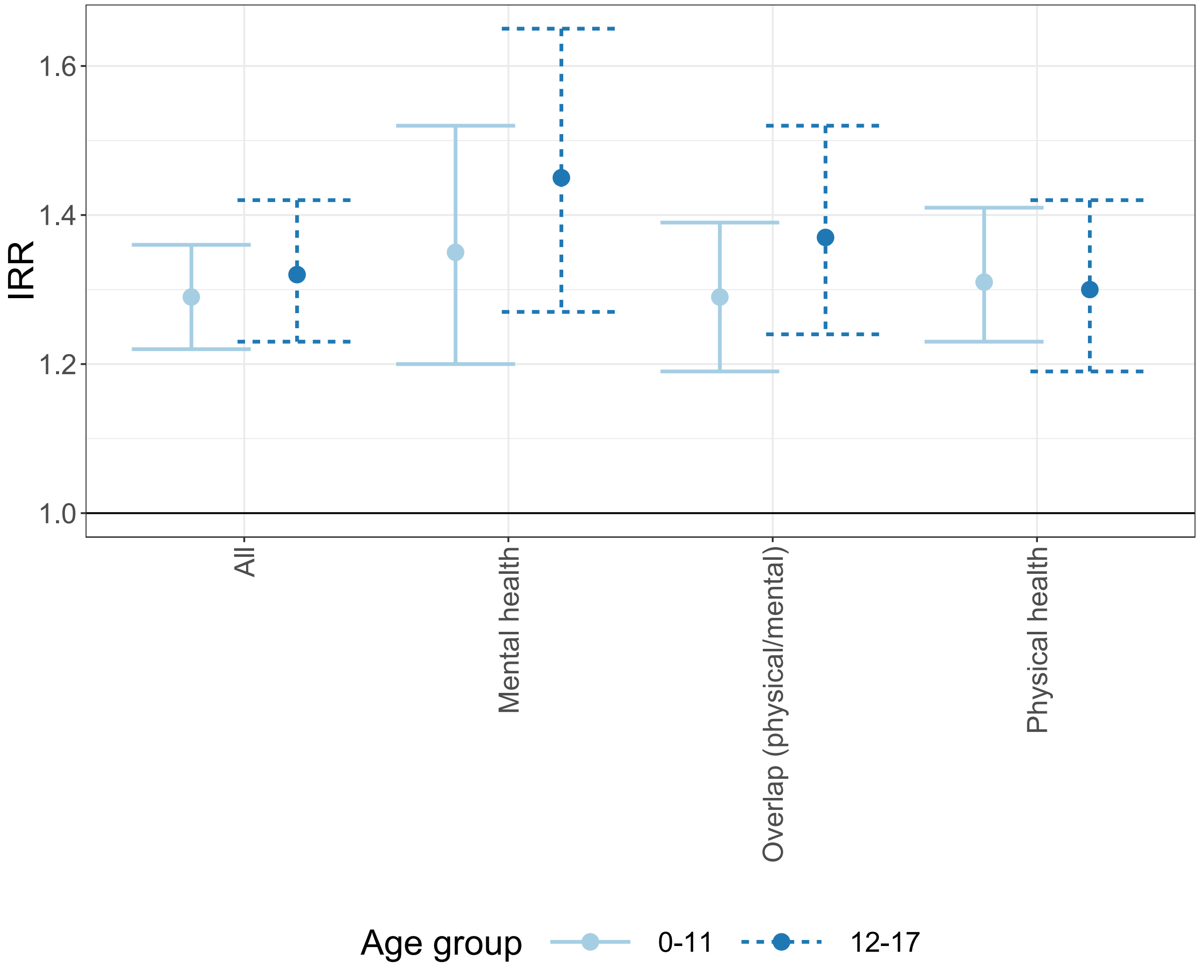

Fig S5: Estimated incidence rate ratios with 95%-confidence intervals in age groups 0-11 and 12-17 by domain
IRR = incidence rate ratio

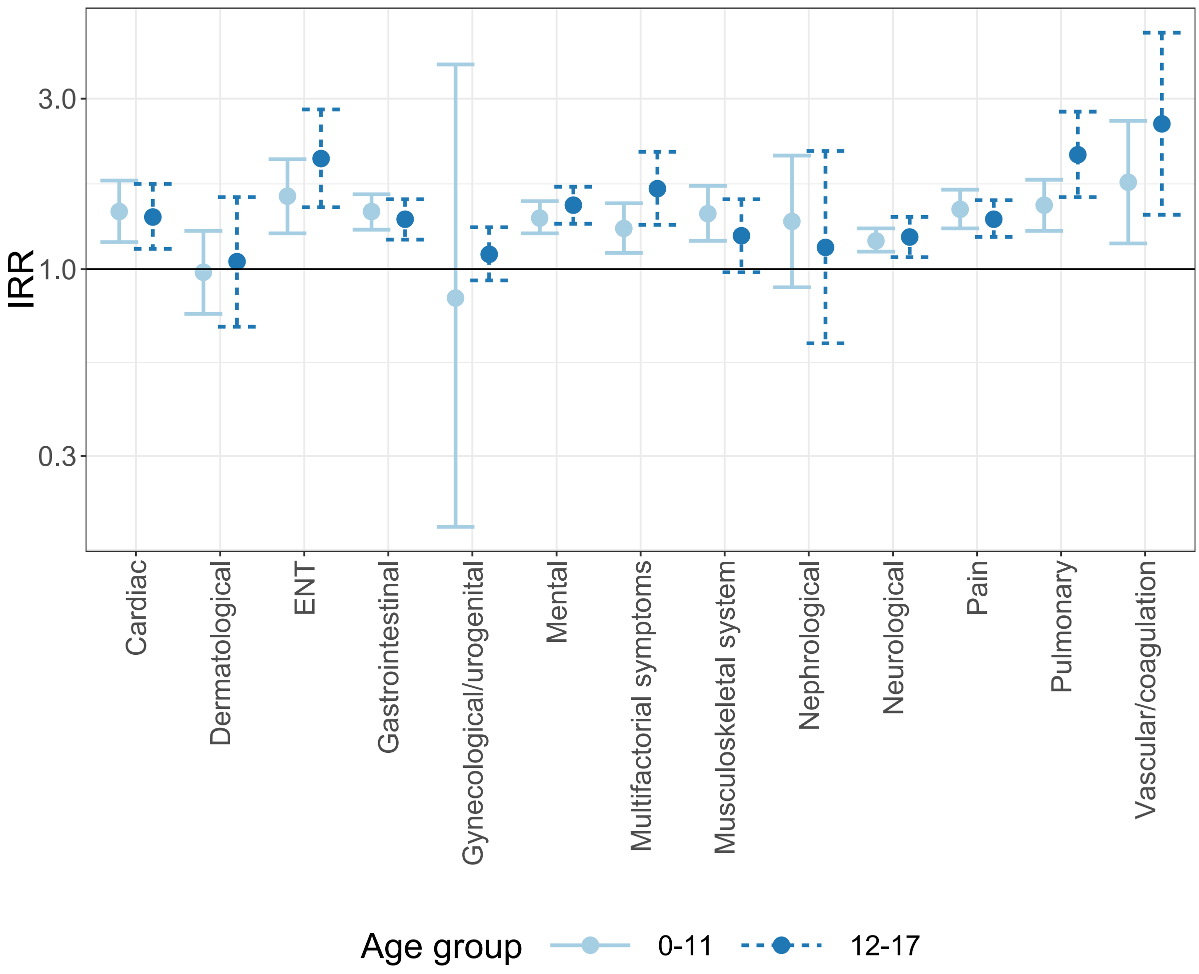

Fig S6: Estimated incidence rate ratios with 95%-confidence intervals in age groups 0-11 and 12-17 by diagnosis/symptom complex
IRR = incidence rate ratio; ENT = Ear, nose and throat

I) Full results for domains and diagnosis/symptom complexes

Table S3: Full results for children and adolescents

Name	n (COVID-19)	IR COVID-19	IR Control	IRR	95%-CI	p
All outcomes	11,950	436.91	335.98	1.30	(1.25-1.35)	<0.01
Mental health outcomes	11,950	102.17	73.24	1.39	(1.28-1.52)	<0.01
Overlap (physical/mental) outcomes	11,950	209.26	158.71	1.32	(1.24-1.40)	<0.01
Physical health outcomes	11,950	254.58	194.45	1.31	(1.24-1.38)	<0.01
Cardiac diagnosis/symptom complex	11,950	37.94	26.55	1.43	(1.24-1.65)	<0.01
Dermatological diagnosis/symptom complex	11,950	12.82	12.82	1.00	(0.80-1.25)	1
ENT diagnosis/symptom complex	11,950	24.86	14.17	1.76	(1.45-2.12)	<0.01
Gastrointestinal diagnosis/symptom complex	11,950	105.28	74.33	1.42	(1.30-1.54)	<0.01
Gynecological/urogenital diagnosis/symptom complex	11,950	22.66	20.62	1.10	(0.93-1.31)	0.28
Mental diagnosis/symptom complex	11,950	127.16	88.21	1.44	(1.33-1.56)	<0.01
Multifactorial symptoms	11,950	44.93	31.78	1.41	(1.24-1.61)	<0.01
Musculoskeletal system diagnosis/symptom complex	11,950	37.94	27.92	1.36	(1.18-1.57)	<0.01
Nephrological diagnosis/symptom complex	11,950	5.96	4.64	1.28	(0.90-1.83)	0.16
Neurological diagnosis/symptom complex	11,950	163.03	134.62	1.21	(1.13-1.29)	<0.01
Pain diagnosis/symptom complex	11,950	104.11	73.09	1.42	(1.31-1.55)	<0.01
Pulmonary diagnosis/symptom complex	11,950	42.21	25.54	1.65	(1.43-1.91)	<0.01
Vascular/coagulation diagnosis/symptom complex	11,950	8.93	4.51	1.98	(1.43-2.75)	<0.01

IRR = incidence rate ratio; IR = incidence rate per 1,000 person-years; CI = 95% confidence interval; p-values are derived from Z-tests of incidence rate ratios

Table S4: Full results for adults

Name	n (COVID-19)	IR COVID-19	IR Control	IRR	95%-CI	p
All outcomes	145,184	615.82	464.15	1.33	(1.31-1.34)	<0.01
Mental health outcomes	145,184	215.62	169.50	1.27	(1.25-1.29)	<0.01
Overlap (physical/mental) outcomes	145,184	278.58	192.59	1.45	(1.42-1.47)	<0.01
Physical health outcomes	145,184	422.87	304.42	1.39	(1.37-1.41)	<0.01
Cardiac diagnosis/symptom complex	145,184	144.64	99.20	1.46	(1.43-1.49)	<0.01
Dermatological diagnosis/symptom complex	145,184	23.96	14.40	1.66	(1.58-1.76)	<0.01
ENT diagnosis/symptom complex	145,184	67.05	42.14	1.59	(1.54-1.64)	<0.01
Gastrointestinal diagnosis/symptom complex	145,184	144.09	99.44	1.45	(1.42-1.48)	<0.01
Gynecological/urogenital diagnosis/symptom complex	145,184	51.41	46.42	1.11	(1.07-1.14)	<0.01
Mental diagnosis/symptom complex	145,184	275.79	206.30	1.34	(1.32-1.36)	<0.01
Multifactorial symptoms	145,184	94.74	69.00	1.37	(1.34-1.41)	<0.01
Musculoskeletal system diagnosis/symptom complex	145,184	70.81	54.81	1.29	(1.25-1.33)	<0.01
Nephrological diagnosis/symptom complex	145,184	20.69	14.83	1.39	(1.32-1.47)	<0.01
Neurological diagnosis/symptom complex	145,184	202.23	148.79	1.36	(1.34-1.38)	<0.01
Pain diagnosis/symptom complex	145,184	186.04	129.71	1.43	(1.41-1.46)	<0.01
Pulmonary diagnosis/symptom complex	145,183	83.34	31.81	2.62	(2.53-2.71)	<0.01
Vascular/coagulation diagnosis/symptom complex	145,184	71.73	57.73	1.24	(1.21-1.28)	<0.01

IRR = incidence rate ratio; IR = incidence rate per 1,000 person-years; p-values are derived from Z-tests of incidence rate ratios

J) Comparison of IRRs for health outcomes since index date and in post COVID-19 phase

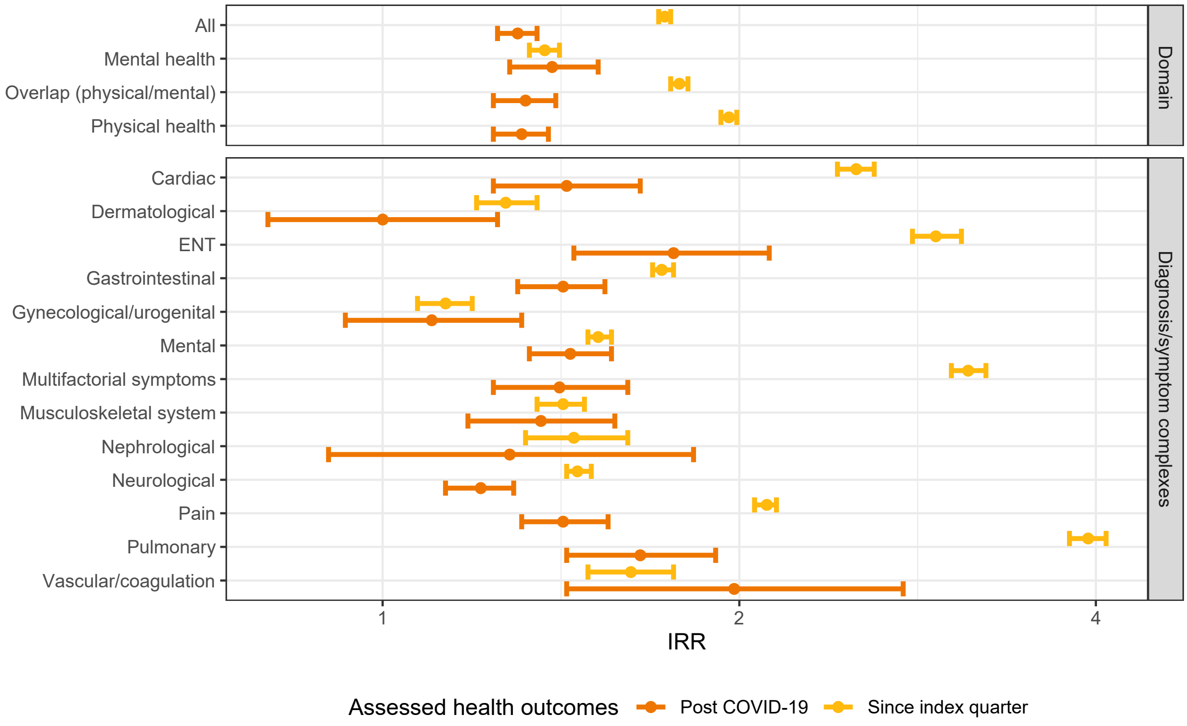

Fig S7: IRRs <3 months after COVID-19 diagnosis and in post COVID-19 phase in childen and adolescents
IRR = incidence rate ratio; ENT = Ear, nose and throat; estimation results are shown on the log-scale

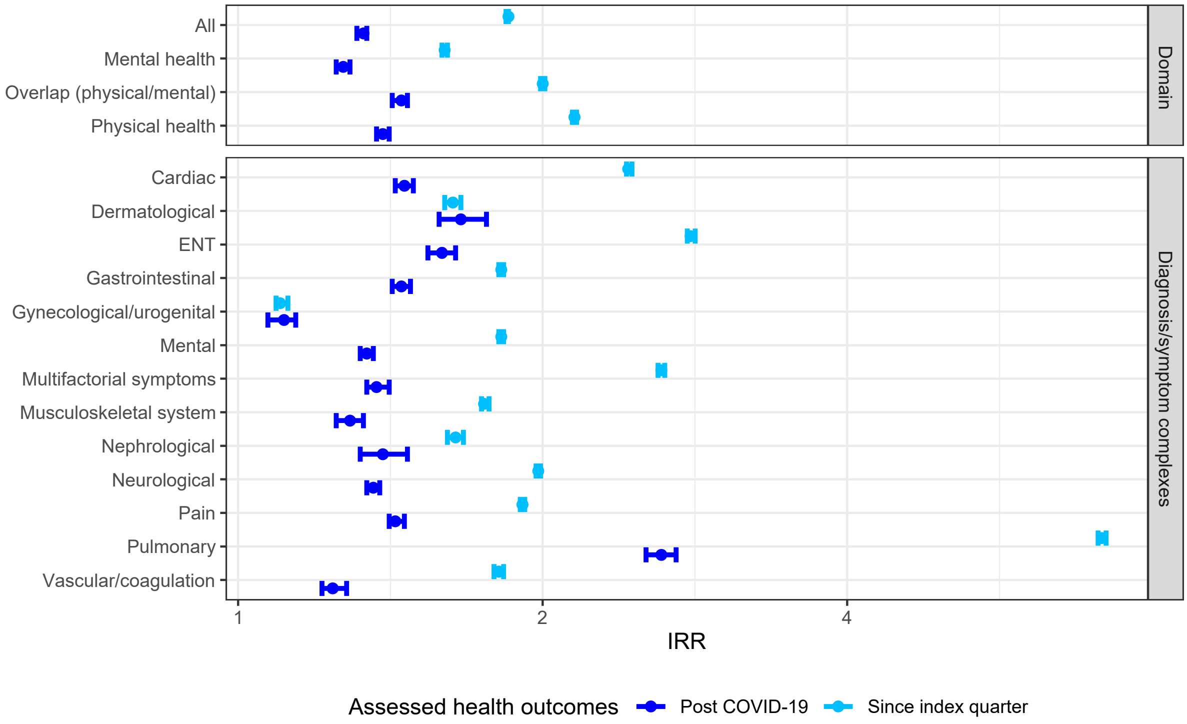

Fig S8: IRRs <3 months after COVID-19 diagnosis and in post COVID-19 phase in adults
IRR = incidence rate ratio; ENT = Ear, nose and throat; estimation results are shown on the log-scale

K) Additional adjustment for healthcare utilization preceding the index date

Table S5: Percentages of hospitalizations and quarters with physician visits in COVID-19 and control cohort

Age group	Variable	Category	COVID-19	Control
Adults	Hospitalization	no	79.7	82.0
		yes	20.3	18.0
	Quarters with physician visit	0	3.4	7.0
		1	5.9	7.7
		2	9.6	10.5
		3	16.5	15.8
		4	64.5	58.9
Children and adolescents	Hospitalization	no	85.7	89.5
		yes	14.3	10.5
	Quarters with physician visit	0	3.8	6.3
		1	7.4	11.6
		2	14.7	17.9
		3	23.8	25.3
		4	50.3	38.8

Table S6: Results for children and adolescents under additional adjustment for healthcare utilization before index date

Name	IRR	95%-CI	p
All outcomes	1.23	(1.17-1.29)	<0.01
Mental health outcomes	1.29	(1.16-1.44)	<0.01
Overlap (physical/mental) outcomes	1.24	(1.16-1.34)	<0.01
Physical health outcomes	1.23	(1.15-1.32)	<0.01
Cardiac diagnosis/symptom complex	1.37	(1.15-1.63)	<0.01
Dermatological diagnosis/symptom complex	0.94	(0.71-1.23)	0.64
ENT diagnosis/symptom complex	1.64	(1.30-2.07)	<0.01
Gastrointestinal diagnosis/symptom complex	1.34	(1.20-1.49)	<0.01
Gynecological/urogenital diagnosis/symptom complex	1.07	(0.87-1.33)	0.51
Mental diagnosis/symptom complex	1.34	(1.21-1.47)	<0.01
Multifactorial symptoms	1.31	(1.12-1.54)	<0.01
Musculoskeletal system diagnosis/symptom complex	1.23	(1.04-1.46)	0.02
Nephrological diagnosis/symptom complex	1.14	(0.75-1.75)	0.54
Neurological diagnosis/symptom complex	1.13	(1.04-1.22)	<0.01
Pain diagnosis/symptom complex	1.35	(1.21-1.50)	<0.01
Pulmonary diagnosis/symptom complex	1.53	(1.28-1.82)	<0.01
Vascular/coagulation diagnosis/symptom complex	1.89	(1.27-2.82)	<0.01

IRR = incidence rate ratio; ENT = Ear, nose and throat; p-values are derived from Z-tests of incidence rate ratios

Table S7: Results for adults under additional adjustment for healthcare utilization before index date

Name	IRR	95%-CI	p
All outcomes	1.26	(1.24-1.27)	<0.01
Mental health outcomes	1.19	(1.17-1.21)	<0.01
Overlap (physical/mental) outcomes	1.37	(1.34-1.39)	<0.01
Physical health outcomes	1.31	(1.29-1.32)	<0.01
Cardiac diagnosis/symptom complex	1.35	(1.32-1.38)	<0.01
Dermatological diagnosis/symptom complex	1.57	(1.47-1.68)	<0.01
ENT diagnosis/symptom complex	1.48	(1.42-1.54)	<0.01
Gastrointestinal diagnosis/symptom complex	1.38	(1.34-1.41)	<0.01
Gynecological/urogenital diagnosis/symptom complex	1.07	(1.03-1.11)	<0.01
Mental diagnosis/symptom complex	1.25	(1.23-1.28)	<0.01
Multifactorial symptoms	1.26	(1.22-1.30)	<0.01
Musculoskeletal system diagnosis/symptom complex	1.17	(1.13-1.21)	<0.01
Nephrological diagnosis/symptom complex	1.28	(1.19-1.36)	<0.01
Neurological diagnosis/symptom complex	1.26	(1.23-1.28)	<0.01
Pain diagnosis/symptom complex	1.36	(1.33-1.39)	<0.01
Pulmonary diagnosis/symptom complex	2.46	(2.36-2.56)	<0.01
Vascular/coagulation diagnosis/symptom complex	1.12	(1.09-1.16)	<0.01

IRR = incidence rate ratio; ENT = Ear, nose and throat; p-values are derived from Z-tests of incidence rate ratios
